# Reovirus infection induces transcriptome-wide unique A-to-I editing changes in the murine fibroblasts.

**DOI:** 10.1016/j.virusres.2024.199413

**Published:** 2024-06-13

**Authors:** Ayesha Tariq, Helen Piontkivska

**Affiliations:** a Department of Biological Sciences, Kent State University, Kent, OH, USA; b Brain Health Research Institute, Kent State University, Kent, OH, USA; c Healthy Communities Research Institute, Kent State University, Kent, OH, USA

**Keywords:** Rna editing, Adar, Reovirus, Interferon stimulation, A-to-i editing

## Abstract

The conversion of Adenosine (A) to Inosine (I), by Adenosine Deaminases Acting on RNA or ADARs, is an essential post-transcriptional modification that contributes to proteome diversity and regulation in metazoans including humans. In addition to its transcriptome-regulating role, ADARs also play a major part in immune response to viral infection, where an interferon response activates interferon-stimulated genes, such as ADARp150, in turn dynamically regulating host-virus interactions. A previous report has shown that infection from reoviruses, despite strong activation of ADARp150, does not influence the editing of some of the major known editing targets, while likely editing others, suggesting a potentially nuanced editing pattern that may depend on different factors. However, the results were based on a handful of selected editing sites and did not cover the entire transcriptome. Thus, to determine whether and how reovirus infection specifically affects host ADAR editing patterns, we analyzed a publicly available deep-sequenced RNA-seq dataset, from murine fibroblasts infected with wild-type and mutant reovirus strains that allowed us to examine changes in editing patterns on a transcriptome-wide scale. To the best of our knowledge, this is the first transcriptome-wide report on host editing changes after reovirus infection. Our results demonstrate that reovirus infection induces unique nuanced editing changes in the host, including introducing sites uniquely edited in infected samples. Genes with edited sites are overrepresented in pathways related to immune regulation, cellular signaling, metabolism, and growth. Moreover, a shift in editing targets has also been observed, where the same genes are edited in infection and control conditions but at different sites, or where the editing rate is increased for some and decreased for other differential targets, supporting the hypothesis of dynamic and condition-specific editing by ADARs.

## Highlights

• Reovirus infections primarily occur in infants and young children, with various respiratory, gastrointestinal, and central nervous system symptoms, and may lead to severe meningitis and encephalitis. This study examines the host-side transcriptome-wide consequences of reovirus infection, in order to better understand the impact of viral infection on the host.
• Our results show that reovirus infection induces transcriptome-wide RNA editing changes, resulting in nuanced and dynamic shifts in the global editing landscape. These shifts include site-specific unique editing changes, particularly in genes involved in cellular regulation and immunity.
• Majority of identified editing changes were observed within 3′UTRs, where these variants can potentially influence gene expression through interruption of micro-RNA binding, polyadenylation, and posttranscriptional processing.

## List of abbreviations

ADAR, Adenosine Deaminases Acting on RNA
APOBECs, Apolipoprotein B mRNA-editing enzyme, catalytic polypeptide
CI, Confidence Interval
ISG, Interferon Stimulated Gene
muReoV, Mutant Reovirus
PKR, Protein Kinase R
ReoV, Reovirus
UTR, Untranslated Region

Reoviruses (ReoV), first isolated from human stool by Sabin in 1950ies (Sabin, 1959), and ubiquitously present in untreated sewage water and stool (Danthi et al., 2013; Lemay 2018), are non-enveloped double-stranded (ds) RNA viruses that belong to the family Spinareoviridae (Forrest and Dermody, 2003; Matthijnssens et al., 2022). DsRNA genome of reoviruses is segmented, encoding a total of twelve proteins, all of which are translated from a positive genomic strand (Dermody et al., 2022). There are four genetically distinct reovirus serotypes, of which type 3 Dearing (T3D) (Dermody et al., 2022; DeAntoneo et al., 2022) is oncolytic (Mohamed et al., 2020; Lin et al., 2023) and can infect humans and mice (Cashdollar et al., 1985; Norman and Lee, 2000; Gauvin et al., 2013).

Reovirus infection most commonly occurs in infants and young children and shows respiratory, gastrointestinal, and central nervous system (CNS) manifestations (Kapikian and Shope 1996; DeBiasi and Tyler, 2015). Although reoviruses are known to cause mostly mild disease in humans, viral strains have been isolated from children with severe meningitis and encephalitis, indicating neurovirulence (Ouattara, 2011). A study in mice showed that the T3D strain causes lethal encephalitis in young mice with no distinct signs of infection in older ones (Wu et al., 2018). A novel strain isolated in Japan from children with meningitis and gastroenteritis was found to be genetically similar to a strain identified from bats in China, suggesting potential interspecies transmission (Yamamoto et al., 2020).

After the virus infects the host cell, viral dsRNA is detected by the host's cytosolic receptors leading to the induction of type I interferon (IFN) response and ultimately the expression of interferon-stimulated genes (ISGs) to stop viral replication (Lanoie et al., 2019; Lemay 2018; Dermody et al., 2022). Reovirus infection also elicits type III interferon response, although the mechanism remains poorly understood (Abad and Danthi, 2020; Abad and Danthi, 2022). Replication of reovirus inside the host cell is inhibited by interferon-inducible protein-dependent kinase or PKR, and reoviral protein σ3 competes with PKR for binding to dsRNA and confers a partial resistance to PKR (Lanoie et al., 2019). For a detailed description of the reovirus multiplication cycle and interferon signaling, the reader is referred to a recent review by Lemay (2018) and Lanoie et al. (2019).

Reoviruses such as T3D can preferentially replicate and survive inside the cancerous cells due to lower induction of IFNs and decreased PKR activity (Phillips et al., 2018). A reovirus strain P4L-12 generated through chemical mutagenesis of wild-type T3D shows hypersensitivity to interferons with a 1000-fold decrease in viral titer inside interferon-treated cells compared to wild-type (Rudd and Lemay, 2005). A study comparing the P4L-12 mutant and T3D strain has shown that the mutant strain was more effectively blocked by interferons in L929 cells (Sandekian and Lemay, 2015). All these factors make reoviruses important candidates to study host virus interactions.

As described earlier, infection of reovirus (ReoV) is known to induce a strong IFN response (Hood et al. 2014; Lanoie et al., 2019; Dermody et al., 2022), in turn activating Adenosine Deaminases Acting on RNA (ADARs) as a downstream consequence of the IFN activation pathways (Pfaller et al. 2021; Samuel, 2011). ADARp150, a long isoform of ADAR1, is an ISG and is present in both the cytoplasm and nucleus while the short isoform p110 is predominantly localized in the nucleus (Pfaller et al., 2021). ADAR1 is known to edit viral RNA, rendering it destabilized for replication. However, the editing is dynamic and can be pro- or antiviral depending on the type of virus (Piontkivska et al., 2021; Tang et al., 2015). Studies from ADAR1/2 knockouts found no impact on ReoV replication (Ward et al., 2011), potentially because of the protective core-like sub-virion particle that shields viral genomes during replication (Pfaller et al., 2021). Studies of animal reoviruses, such as that of grass carp reovirus (GCRV), showed activation of an ADAR1 homolog in response to viral infection (Yang et al., 2012; Rao and Su, 2015), implying a role of the enzyme in the antiviral response, although its impact on the viral editing remains to be elucidated.

It has been established that RNA viruses, including reoviruses, experience ADAR editing during infection (eg, Tang et al., 2015; Pfaller et al., 2021; Piontkivska et al., 2021; Ringlander et al., 2022; Zhu et al., 2023). However, the impact of viral infection on the editing of the host transcriptome is not well understood (Piontkivska et al., 2021). A study from neonatal mice injected with reovirus T3D into the brain found that despite strong induction of ADARp150, the majority of surveyed editing substrates exhibited little to no editing changes, including no detectable changes within *5HT2C* (serotonin 2C receptor) transcripts (Hood et al., 2014). Nonetheless, some genes did experience changes in editing, such as cytoplasmic FMR1 interacting protein 2 (*Cyfip2*), filamin A (*Flna*), and bladder cancer-associated protein (*Blcap*) (Hood et al., 2014), indicating that infection-induced changes in editing patterns may be nuanced. Thus, to better understand the effect of reovirus infection on changes in ADAR editing of the host (if any) at a whole-transcriptome scale, we took advantage of a publicly available deep-sequenced BioProject PRJNA320288 dataset, from Boudreault et al., 2016 study (GEO accession number GSE81017), where the L929 mice cell lines were infected with wild-type and mutant ReoV strains, and compared their ADAR editing patterns to those of the uninfected control cells (Table 1).

**Table 1:** Characteristics of the BioProject (PRJNA320288) dataset used in the study. ReoV infected represents fibroblasts (L929) cells infected with wild-type reovirus (ReoV) T3D, while muReoV denotes cells infected with mutant reovirus (muReoV) P4L-12. Total RNA was extracted 14 h after infection (Boudreault et al., 2016). The total number of edited sites represent total edited sites with editing rate > 0.01 & < 0.99, but not between 0.49 & 0.51, in each condition. Mean Alu Editing Index (AEI) was calculated by taking the average of A-to-G indices for all samples in one condition. Assembly ASM629838v1 was used as ReoV reference genome.

Experimental treatment	Infection period	Sample Count	Sample ID	Total number of reads	Percent of reads mapped to GRCm38	Percent of reads mapped to ReoV	Total number of edited sites	Total number of edited sites with reference in REDI portal	Mean A-to-G Alu Editing Index (AEI)
Control	0hrs	3	SRR3471108	92,700,662	88.99 %	0.00 %	487	17	0.260720
			SRR3471109	94,009,468	88.95 %	0.00 %			
			SRR3471110	78,807,536	89.09 %	0.00 %			
ReoV (T3D) Infected	14hrs	3	SRR3471111	103,529,228	87.83 %	0.63 %	784	71	0.286890
			SRR3471112	104,993,674	87.76 %	0.62 %			
			SRR3471113	88,509,976	87.81 %	0.63 %			
muReoV (P4L-12) Infected	14hrs	3	SRR3471114	154,880,632	87.36 %	0.73 %	1284	153	0.31529
			SRR3471115	157,190,906	87.30 %	0.73 %			
			SRR3471116	131,616,182	87.32 %	0.73 %			

The Automated Isoform Diversity Detector (AIDD) pipeline (Plonski et al., 2020) was used to map the reads, variant calling, and predict ADAR editing sites (Supplemental_Table_1). DESeq2 R package (Love et al., 2014) was used for differential expression analysis of ADAR genes and transcripts. The Alu Editing Index (AEI) as a global measure of editing was calculated per sample using the RNAEditingIndexer tool (Roth et al., 2019). Briefly, the editing index is calculated as the ratio of the number of A-to-G mismatches to the total coverage of the adenosine sites that encompasses both A-to-A matches and A-to-G mismatches at the repetitive elements, such as SINE elements, in mouse (Neeman et al., 2006; Roth et al., 2019). Variant frequency counts estimated using bam-readcount (Khanna et al., 2022) were used to calculate the average editing rate per site. The editing rate was defined as the number of G reads for an A reference site or C reads for a U(T) reference site on an opposite strand, divided by the total number of reads mapped to that site. Average editing was calculated by taking the mean of editing rate for a single site across all samples for a specific condition. Control samples were compared with infected samples for differential editing changes. A similar comparison was made for samples infected with wild-type vs mutant strains. To ensure that only confirmed editing sites are considered, only those editing sites with reference in the REDIportal, a comprehensive nonredundant database of A-to-I editing events (Picardi et al., 2017), were included. Other filtering options required that the edited site had greater than or equal to 20 total reads aligned, and that editing rates were greater than 0.01, or less than 0.99, and not between 0.49 and 0.51, to remove potential noise, homozygous genomic variants, and heterozygous genomic variants, respectively.

We performed differential editing analysis for all the edited sites, independent of their absence or presence in REDIportal (Supplemental_Files_15&16). Annotations for those sites were obtained from SnpEff (Cingolani et al., 2012) incorporated in to the AIDD pipeline. To visualize alternatively spliced isoform, we made Sashimi plots using Integrative genomics viewer or IGV (Robinson et al., 2023) with minimum junction coverage of 5 reads, according to GenVision Pro-17.3.1, user manual (Sashimi tracks, 2024).

Pathway enrichment analysis was done using The Database for Annotation, Visualization, and Integrated Discovery (DAVID) functional annotation tool (Huang et al., 2009; Sherman et al., 2022). R package biomaRt (Durinck et al., 2009) was used for annotation of differentially expressed genes. We used the Ensembl Variant Effect Predictor (VEP) to analyze the potential impact of novel A-to-I edits on the transcript (McLaren et al., 2016). R packages ggplot2 (Wickham, 2016), ggrepel (Slowikowski, 2023), ggpubr (Kassambara, 2018), tidyr (Wickham et al., 2023c), dplyr (Wickham et al., 2023b), readr (Wickham et al., 2023a), stringr (Wickham, 2019) and a web-based tool InteractiVenn (Heberle et al., 2015) were used for statistical analysis and data visualization.

**Fig. 1:**
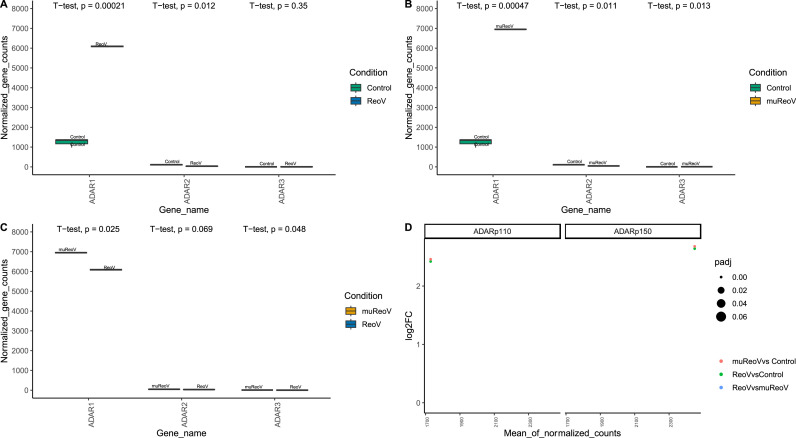
Differences in expression of ADAR genes, and distribution of ADAR editing between ReoV-infected, muReoV-infected, and uninfected control samples. Normalized count values from DESeq2 are plotted here to show the differential expression of ADAR genes and transcripts. All ADAR genes except ADAR3 are significantly differentially expressed in ReoV_vs_Control (A). While all three ADARs show significant differential expression in muReoV_vs_Control (B), in muReoVvsReoV (C) difference in ADAR1 and ADAR3 expression is significant. ADARp150, and ADARp110, are significantly differentially expressed in all comparisons (D) except ReoV_vs_muReoV.

The expression of all three ADAR genes was taken as normalized gene counts from DESeq2 (Fig. 1, Supplemental_Table_2C-2D). As shown in Fig. 1, ADAR1 is significantly highly expressed in ReoV (Fig. 1A) and muReoV (Fig. 1B) samples as compared to control, while ADAR1, expression is significantly different between ReoV and muReoV samples (*p* = 0.025) as well (Fig. 1D). The normalized count values for ADAR2 and ADAR3 are relatively low; nonetheless, ADAR2 expression differs significantly between control and ReoV, and control and muReoV (*p* = 0.012, 0.011, respectively) while ADAR3 differs between control and muReoV (*p* = 0.013), and between ReoV and muReoV (*p* = 0.048).

**Fig. 2:**
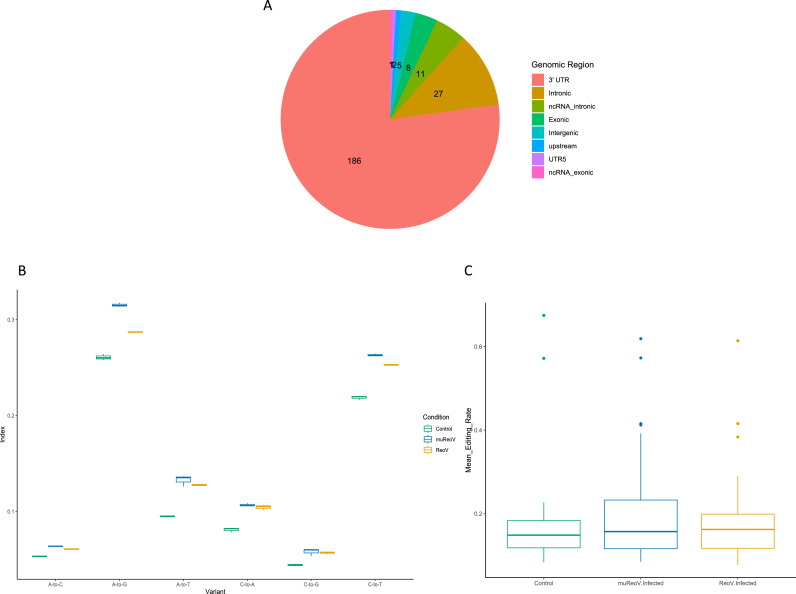
Figure shows the number of region-specific edits, global A-to-G editing index and mean editing per sample. Panel A shows the number of region-specific editing changes with an editing rate > 0.01 in all samples. Panel B shows a comparison of the Alu Editing Index (AEI) (Roth et al., 2019) for all three conditions. The A-to-G editing index is higher in infected samples. Panel C shows mean editing rate per site per sample for all three conditions. More sites are edited in infected samples, with the highest editing rate in muReoV infection.

Differential expression analysis with DESeq2 confirmed these findings, where the expression of ADARp110 and ADARp150 transcripts was significantly higher, more than two-folds, in both ReoV- and muReoV- infected samples compared to controls, with padj values < 0.05 (Fig. 1D, Supplemental_Table_3 & Supplemental_Table_4). However, at the gene level only ADAR1, but not ADAR2 or ADAR3, was among differentially expressed genes (DEGs) (Supplemental_Table_4A) for the ReoV-vs-control comparison, with log2FoldChange at ∼2.48 (padj values < 0.05), while ADAR2 was among DEGs for the muReoV-vs-control comparisons, respectively, although the fold change was much smaller, at ∼ 1.12 (padj values < 0.05) (Supplemental_Table_4C). Next, we examined region-specific editing and differential editing per site. The highest number of edited sites falls in 3′UTRs, while 5′UTRs are the least edited, among all edited genomic regions (Fig. 2A). The Alu Editing Index (AEI), for A-to-G changes, is also high for infected samples, with C-to-U(T) being the next most common transition (Fig. 2B, Supplemental_Table_5A), reflecting infection-triggered changes in ADAR editing activity (Roth et al., 2019). We further examined whether the reads unmapped to the mouse reference genome represent hyper-edited viral or murine reads, using RNA hyper-editing pipeline from Porath et al. (2014) with default parameters (Supplemental_Table_5B and Supplemental_Table_5C, respectively). Although unique A-to-G changes were present, no hyper-editing among viral reads was detected after the applied quality control filters (Supplemental_Table_5B). This can be attributed to ADAR editing mostly targeting host rather than viral transcripts in early infection, similar to the pattern reported in herpesvirus 1 infection in Crassostrea gigas oysters (Rosani et al., 2022). However, despite the majority of potentially hyper-edited A-to-G changes being mapped to murine rather than viral genome (Supplemental_Table_5C), those reads also did not pass the quality control filters. Thus, lack of detected hyper-editing here may reflect a highly nuanced nature of ADAR editing, and that of hyper-editing in particular, where levels and targets of the latter phenomenon vary with the virus, stage of infection and host genetic factors (Ivanišević et al., 2023).

**Fig. 3:**
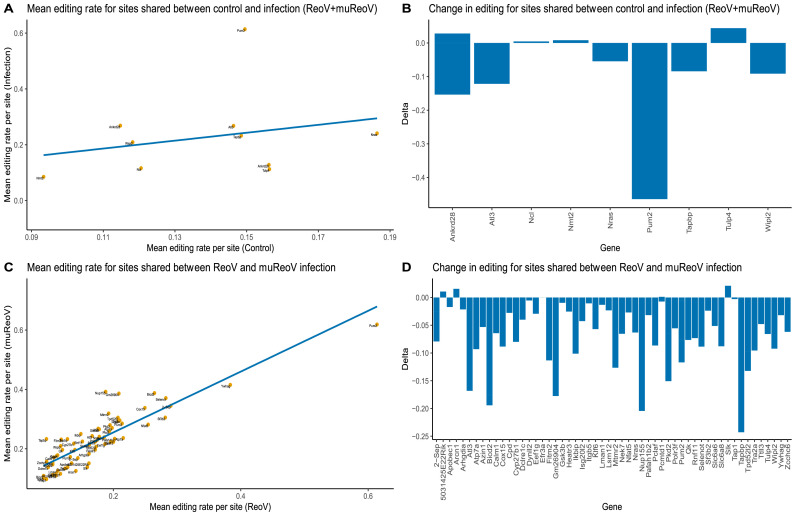
Difference in mean editing rate, per site, between control and infected samples. Only sites present in REDIportal are shown. Panel A represents mean editing for sites common across all control and infected samples. The grey area represents 95 % CI for the difference in editing. Panel C shows mean editing for sites common in muReoV and ReoV only. Panels B and D show the difference in mean editing per site for control vs infection (ReoV + muReoV) and ReoV vs muReoV only, respectively. The full list of edited sites is available in Supplemental_Table_8 and Supplemental_Table_9. For panels, B and D, a negative delta value represents an increased editing rate in muReoV samples.

For the initial comparison, we focused on all edited sites, including those that did not map to REDIportal genes. The results showed that there is a shift in editing between control, ReoV infected, and muReoV infected samples, where infected and mutant infected samples show an increase in the number of editing sites (Fig. 2C). Overall, control, reovirus T3D or ReoV infected and mutant P4L-12 or muReoV infected samples had 487, 784, and 1284 A-to-G or U(T)-to-C edited positions respectively (Supplemental_Figure_1, Supplemental_File_15), where 231 sites were shared between all samples, 249 between ReoV and control, 288 between muReoV and control, and 554 between muReoV and ReoV (Supplemental_File_16). Next, all the A-to-G or U(T)-to-C substituted sites were filtered for those mapped to REDIportal genes. Only 17 sites were mapped to genes from control samples while in wild-type (ReoV) and mutant reovirus-infected samples (muReoV), 71 and 153 sites were mapped to REDIportal genes, respectively (Supplemental_Figure_2, Supplemental_Table_7). Overall, only 9 positions were in common between infection and control, with 6 positions shared across all three conditions, with significant differential editing for a few positions. Mean editing of common sites was higher in infection than in control, with the PUM2 gene experiencing the highest amount of change (Fig. 3B, Supplemental_Table_8). Similarly, 66 sites among sites unique to infection samples were shared between ReoV and muReoV infection, and the overall editing rate was higher in muReoV samples, with the highest mean editing in the Tapbp gene (Fig. 3D, Supplemental_Table_9). Moreover, 87 positions were only edited in mutant-infected samples (Supplemental_Table_10), while only 5 positions were unique to ReoV-infected samples (Supplemental_Table_11).

**Fig. 4:**
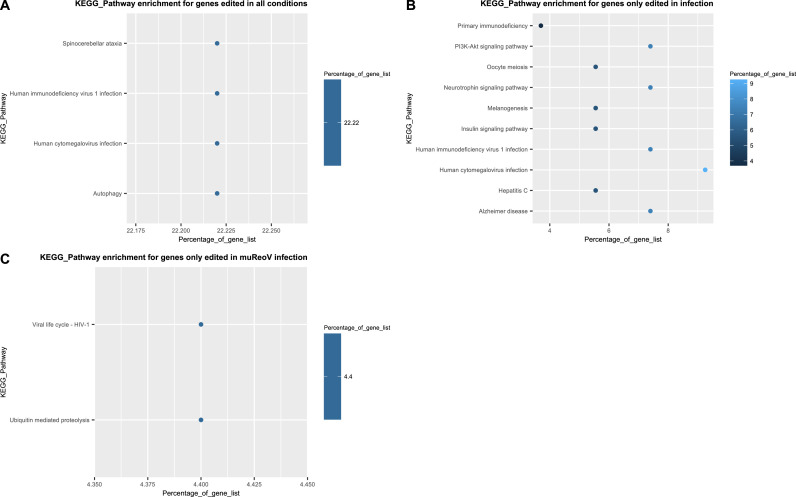
KEGG pathway enrichment for sites in all treatment conditions and unique to infection samples. Here, infection represents sites absent in control but shared between ReoV and muReoV. Panels A, B, and C show pathway enrichment for genes edited in all conditions (A), shared in infection (B), and unique to muReoV (C), respectively.

Our results from pathway enrichment analysis show a shift in affected pathways from control to infection (Fig. 4). Not only different pathways are affected in infected samples compared to control, but editing in infected samples also appears to be spread across a larger number of pathways than that of control samples (Fig. 4B). Gene-specific descriptions of expanded biological categories from InnateDB (Lynn et al., 2008; Lynn et al., 2010; Breuer et al., 2013) can be found in Supplemental_Table_12.

One of the many important components of the host-virus interaction dynamics is the editing of viral as well as host RNA by host ADARs (Piontkivska et al., 2021). Our results demonstrate that the reovirus infection induces nuanced changes in the ADAR editing pattern of the host transcriptome. Reovirus infection causes an increase in the ADAR1 expression that translates to editing changes that are not unidirectional but dynamic, where some sites may experience a consistent increase in editing in infection, while other sites experience a consistent decrease. Genes from our analysis that experienced ADAR editing participate in cellular metabolism, growth, and immune function pathways. Interestingly, edited sites in muReoV infected samples showed enrichment in genes from the ubiquitin-mediated proteolysis (Fig. 4C). While further studies are needed, this enrichment may be related to viral exploitation of ubiquitin-mediated proteolysis machinery to target anti-viral proteins, hence enhancing their survival (Luo, 2016).

We also have observed a shift in editing targets, where the same gene is being edited in control and infection, but at a different position (Supplemental_Table_8B). For instance, the Atl3 gene associated with cellular integrity and cytokinesis (Mohammadi et al., 2023) has only one edited site in 3′UTR at position 7,538,115 in control, while positions 7,535,531 and 7,538,115, are edited in both muReoV and ReoV, and position 7,535,531 is edited only in muReoV infected samples. Similarly, in Rpa1 gene 3′UTR is edited at position 7,530,101 in control while the editing targets shifted to positions, 75,300,618, 75,300,622, 75,300,985, and 75,301,000 in muReoV infected samples. One explanation for that phenomenon could be that RNA editing is condition-specific (Liew et al., 2017), and/or reflects the effect of other unknown factors that regulate host-virus interactions (Piontkivska et al., 2021). Likewise, editing patterns may also vary depending upon both viral and host genetics (Gelbart et al., 2020).

It is known that reoviruses can preferentially infect and replicate inside Ras-transformed cells, leading to oncolysis (Phillips et al., 2018). Reovirus infection also induces an anti-tumor immune response in cancerous cells, independent of activated Ras signaling, but the exact mechanism is not fully elucidated (Gong et al., 2016; Prestwich et al., 2008; Phillips et al., 2018). It can be speculated that differential ADAR editing is one of the host factors contributing to reovirus-induced anti-tumor immunity. For instance, Pumilio proteins, PUM1 and PUM2, are evolutionarily conserved proteins and have been identified as positive regulators of interferon signaling upon viral infection, by meditating the activation of interferon response factor (IF) and NF-κB (Narita et al., 2014). In our analysis of edited sites unique to infection samples, the PUM2 gene was found to be one of the most edited genes (Table 2), with 3 edited positions in 3′UTR. Moreover, Tapbp gene (Table 2) was only edited in infection samples with increased editing in muReoV infection (Fig. 3D). Tapbp (TAP binding protein) mediates the interaction between MHC and TAP molecules to effectively load and transport antigenic peptides (Boyle et al., 2013). Although the expression of ADAR1 transcripts is not significantly different between ReoV and muReoV (Fig. 1D), ADAR1 normalized gene counts are significantly higher in muReoV (Fig. 1C). Similarly, muReoV samples experienced increased editing (Fig. 2B) and a higher number of differentially edited sites, even though the read coverage for ReoV and muReoV samples is similar (Table 1).

**Table 2:** List of genes with significant ADAR editing sites that are unique to infection samples, along with the region of change, number of edited sites in each gene, and functional relevance of the gene.

Gene	Number of edited sites	Genic location of edited site	Significance of the edited gene
**Arcn1**	1	3′UTR	Involved in vesicle trafficking contain ER synthesized proteins. Mutations in the gene are associated with developmental defects (Izumi et al., 2016)
**Arhgdia**	1	3′UTR	Encodes for Rho GTPase. Deletion in ARHDIA has been found to be associated with congenital nephrotic syndrome (Gupta et al., 2013)
**Apobec1**	1	3′UTR	A, C-to-U deaminase in vertebrates which can act on single-stranded DNA or RNA (Lerner et al., 2018)
**Atl3**	2	3′UTR	Function in the fusion of ER tubules. Mutations associated with hereditary spastic paraplegia and hereditary sensory and autonomic neuropathy (Krols et al., 2019)
**Atp7a**	2	intergenic	Participates in intracellular copper transport. A defective APT7A gene can lead to neurodegeneration (Kaler, 2011)
**Azin1**	1	exonic	Azin1 is one of the best studied ADAR editing targets in cancer in which editing in Exon 12 (consistent with our results) is associated with aggressive tumors (Ghalali et al., 2022; Tay et al., 2021)
**Bicd2**	3	3′UTR	BICD2 is associated with microtubule-based cellular transport and mutations are associated with neural disorders (Luo et al., 2022)
**Calm1**	1	3′UTR	A cellular calcium sensor. A Study has shown that the alternating length of 3′UTR affects CALM1 gene function (Bae et al., 2020)
**Cpd**	1	3′UTR	A transmembrane carboxypeptidase that is involved in protein trafficking (Kalinina et al., 2002)
**Cox15**	1	3′UTR	An oncogene involved in cell proliferation (Zhang et al., 2021)
**Cyp27b1**	1	upstream	Required for vitamin D metabolism. Single nucleotide mutation can result in splicing error (Zou et al., 2020)
**Dynll2**	1	3′UTR	Regulate the activity of protein complexes involved in mitochondrial apoptosis (Singh et al., 2020)
**Dclre1c**	1	3′UTR	Dclre1c codes for an important component of V(D) J pathway that is a prerequisite for antigenic diversity. Mutation in this gene may lead to combined immunodeficiency (Ghadimi et al., 2023)
**Eef1g**	1	intronic	Promotes strain-specific viral replication during host-virus interaction (Sammaibashi et al., 2018)
**Efr3a**	1	3′UTR	Critical for neural synapse synthesis and is one of the GWAS identified risk gene associated with Autism Spectrum Disorder or ASD (Gupta et al., 2014)
**Fitm2**	1	3′UTR	A transmembrane protein, required for lipid homeostasis (Chen et al., 2021)
**Gsk3b**	1	3′UTR	A glycogen synthase kinase with a large number of substrates. GSK3B is involved in important processes such as inflammation and contributes to abnormal phosphorylation of tau protein (Beurel et al., 2015)
**Heatr3**	1	3′UTR	Participates in the nuclear import of newly synthesized ribosomal proteins. Polymorphism in HEATR3 gene is associated with rare cases of anemia (O’Donohue et al., 2022)
**Ikbip**	1	3′UTR	Codes for a kinase interacting protein that promotes apoptosis and plays role in tumor progression (Yang et al., 2021)
**Isg20l2**	1	3′UTR	Serves in biogenesis of ribosomes and can be diagnostic biomarker for breast cancer (Yin et al., 2021; Couté et al., 2008)
**Itgb5**	1	exonic	A transmembrane glycoprotein that can serve as a biomarker for carcinogenesis. Mutations may affect cellular signaling (Liu et al., 2022a)
**Klf6**	1	3′UTR	Regulate transcription in genes lacking TATA promoters and promotes cell proliferation (Dionyssiou et al., 2013)
**Lman1**	1	3′UTR	A cargo receptor for various proteins including (Fu et al., 2019)
**Lsm12**	1	3′UTR	Regulates calcium signaling and RNA splicing. Splice variants of Lsm12 can serve as oral cancer biomarkers (Dong et al., 2022; Zhang et al., 2021)
**Mtmr2**	1	3′UTR	Mutation in MTMR2 gene has been found to be associated with the onset of Charcot–Marie–Tooth neuropathy or CMT (Zambon et al., 2017)
**Nek7**	1	3′UTR	Widely expressed across tissues and is involved in regulating mitosis and activation of inflammasomes (Sun et al., 2020)
**Nfat5**	1	3′UTR	Primarily maintains cellular homeostasis in response to osmotic stress, but can also promote the activation of T cells and macrophages (Lee et al., 2019)
**Nras**	1	3′UTR	An oncogene that exhibits variant specific expression (Eisfeld et al., 2014)
**Nup155**	1	3′UTR	A nucleoporin protein associated with cardiopathy (Leonard et al., 2020)
**Pafah1b2**	1	3′UTR	Regulates platelets activity during important physiological processes such as inflammatory response (Scott et al., 2008)
**Pclaf**	1	3′UTR	A nuclear protein that binds to PCNA, which plays an important role in cell proliferation and synthesis. PCLAF is highly expressed in neuroblastomas (Liu et al., 2022b)
**Pcmtd1**	2	3′UTR	Expressed in eye tissues involved in the pathogenesis of Primary angle-closure glaucoma, with its function largely unknown (Awadalla et al., 2013)
**Pkd2**	2	3′UTR	Responsible for nearly 15 % of autosomal dominant polycystic kidney disease cases, a monogenic disease. Changes in miRNA binding motif in 3′UTR cause a change in gene expression (Lakhia et al., 2022)
**Polr3f**	1	3′UTR	RNA polymerase III subunit F (polfr3) is associated with virus induced encephalitis (Lata et al., 2021)
**Pum2**	3	3′UTR	Regulates interferon signaling through IF and NF-κB (Narita et al., 2014)
**Rnf11**	1	3′UTR	RNF11 modulates ubiquitin-based degradation signaling and is highly conserved among vertebrate (Mattioni et al., 2020)
**Selenot**	1	3′UTR	SELENOT is one of the least studied members of ER based human selenium containing proteins. SELENOT is thought to be required for proper neurodevelopment and its association with PD has been exhibited in mouse models (Varlamova et al., 2022)
**Sept2**	2	3′UTR	Another oncogene associated protein important for cellular organization and growth (He et al., 2019)
**Sf3b2**	1	3′UTR	Contributes to DNA repair through homologous recombination repair (Prados-Carvajal et al., 2018)
**Slc6a6**	1	3′UTR	A neurotransmitter transporter that is highly expressed in colorectal cancer (Yasunaga and Matsumura, 2014)
**Slc6a8**	1	3′UTR	Required for cellular creatin uptake and is associated with X-linked neural disorders (Ghirardini et al., 2023)
**Slk**	1	3′UTR	SLK is a kinase involved in the remodeling of cytoskeleton and cell proliferation (Pryce et al., 2017)
**Tap1**	1	3′UTR	Cytosolic antigen transporter, for MHC class I molecules. Polymorphism in TAP1 and related genes is associated with autoimmune disorders such as Grave's disease (Ritz and Seliger, 2001)
**Tapbp**	2	3′UTR	Involved in MHC class-I associated antigen processing and peptide loading (Teng et al., 2002)
**Tpd52l2**	2	3′UTR	Involved in cell proliferation. Increased expression of the gene has been observed in different carcinomas in association with immunosuppression (Zhong et al., 2021)
**Tra2a**	2	intronic	TRA2a is an RNA binding protein that inhibits splicing of the l RNA as well as regulates host's alternative splicing (Zhao et al., 2021)
**Ttll3**	1	intronic	The only glycylase expressed in humans and could be an effective target to cure Retinitis Pigmentosa (Nazlamova et al., 2022)
**Tulp4**	1	intronic	Member of the tubby-like protein family coordinates multiple signaling pathways such as ubiquitination (Mukhopadhyay and Jackson, 2011)
**Wipi2**	1	3′UTR	Required for autophagy. Mutations in WIPI2 gene are known to cause developmental abnormalities (Kawabata and Yoshimori, 2020)
**Ywhag**	1	3′UTR	Associated with cell signaling and one of the autism risk genes also involved in developmental abnormalities (Ye et al., 2021)
**Zcchc6**	1	intronic	Involved in post transcriptional regulation of pro inflammatory cytokines. Decreased expression of ZCCHC6 leads to increased cytokine production (Kozlowski et al., 2017)

ADAR editing sites are occasionally found near alternative splicing (AS) regions and splice junctions; however, ADAR1 knockout cells showed a global change in AS patterns (Solomon et al., 2013) indicating a complex relationship between editing and splicing. We compared our list of differentially edited genes with the list of 193 differentially spliced gene from the original Boudreault et al. (2016) study. We found multiple genes that had both differential editing and differential splicing (Supplemental_File_17A). However, none of these genes had ADAR editing events in regions directly involved in splicing (defined as splice region, splice donor or acceptor, as per snpEff output for variant type), consistent with the complex regulatory relationship between ADAR editing and AS. Furthermore, in agreement with the Solomon et al. (2013) findings that direct ADAR editing does not play a primary regulatory role in AS, only a small percentage of our differentially edited sites (∼1 %) was found within splice regions (Supplemental_File_17B). Supplemental_File_17C shows Sashimi plots that visualize these newly identified alternative splicing patterns supported by at least 5 reads within differentially edited genes, including alternatively spliced isoforms for HJURP, ANKRD13, ALC4A1AP, and TRIM24 genes.

In conclusion, our analysis of transcriptome-wide RNA editing changes in murine fibroblasts conclusively shows that infection with reoviruses indeed affects the global editing landscape, leading to site-specific unique editing changes. Similar to prior studies, the majority of editing changes observed here are located within 3′ UTRs (e.g., Wales-McGrath et al., 2023), underscoring the need to understand the consequences of such seemingly minor and likely dynamic changes on regulation of gene expression. Variants in 3′UTRs can influence gene expression through interruption of micro-RNA binding, polyadenylation, and posttranscriptional processing (Skeeles et al., 2013); changes in UTRs can also contribute to disease progression by affecting mRNA expression (Hong and Jeong, 2023). We also identified several editing events within splicing-related regions that may be directly involved in regulation of alternative splicing (Supplemental_Files_17A-C), although further studies are needed. Furthermore, to verify that our inference of absence of editing at specific sites is not skewed by lack of reads covering these sites, we examined nucleotide states of sites that experienced editing in a single condition. Our results confirmed that these sites were indeed covered by reads in other conditions, but did not harbor reads with alternative nucleotides; in other words, these sites do experience different editing outcomes (Supplemental_File_18).

The effect of viral infections on the RNA editing patterns of the host remains largely underexplored, with very little if any data available for many viral families (Piontkivska et al., 2021). Future ReoV studies should focus on tissue and tumor-specific patterns of ADAR editing in humans, for a safer reovirus-based oncolytic therapy. Moreover, such studies should also consider changes in editing mediated by APOBECs (Dolan et al., 2018; Piontkivska et al., 2021), given the observed increase in C-to-U(T) index in infected cells (Supplemental_Table_5). Comparison of the host's editing landscape after reovirus infection with related pathogens, such as rotavirus (Crawford et al., 2017), could clarify whether or how ADAR editing is affected by the severity of infection. In our analysis, we also identified 2314 novel A-to-G or U(T)-to-C changes (Supplemental_Table_13) that were not reported in REDIportal. Many of these sites fall in intergenic regions and may have modifier effects on the transcript (Supplemental_File_14). Experimental validation of these sites can offer further insights into the effect of reovirus infection on host transcriptome. While this study is limited by a small sample size, future studies should focus on experimental validation of the reported findings with a larger sample size, to expand our understanding of the interaction of reoviruses with host cells.

### Availability of data and materials

Supplementary Figures and Tables, with relevant input data files and R code, are available at GitHub repository at https://github.com/RNAdetective/Reovirus_editing. The dataset used in this current study is publicly available in the NCBI SRA/BioProject repository, as BioProject PRJNA320288.

### CRediT authorship contribution statement

**Ayesha Tariq:** Writing – review & editing, Writing – original draft, Visualization, Investigation, Formal analysis, Data curation. **Helen Piontkivska:** Writing – review & editing, Writing – original draft, Supervision, Project administration, Methodology, Formal analysis, Data curation, Conceptualization.

## Declaration of competing interest

The authors declare that they have no known competing financial interests or personal relationships that could have appeared to influence the work reported in this paper.

## Data Availability

Data and code are shared at https://github.com/RNAdetective/Reovirus_editing.

## Appendix Supplementary materials

Image, application 1

Image, application 2

Image, application 3

Image, application 4

Image, application 5

Image, application 6

Image, application 7

Image, application 8

Image, application 9

Image, application 10

Image, application 11

Image, application 12

Image, application 13

Image, application 14

Image, application 15

Image, application 16

Image, application 17

Image, application 18

Image, application 19
