# Supplementary figures and images for: Reovirus infection induces transcriptome-wide unique A-to-I editing changes in the murine fibroblasts
Source: Virus Res. 2024 Jun 13;346:199413. doi: 10.1016/j.virusres.2024.199413 (PMC11225029; doi:10.1016/j.virusres.2024.199413)

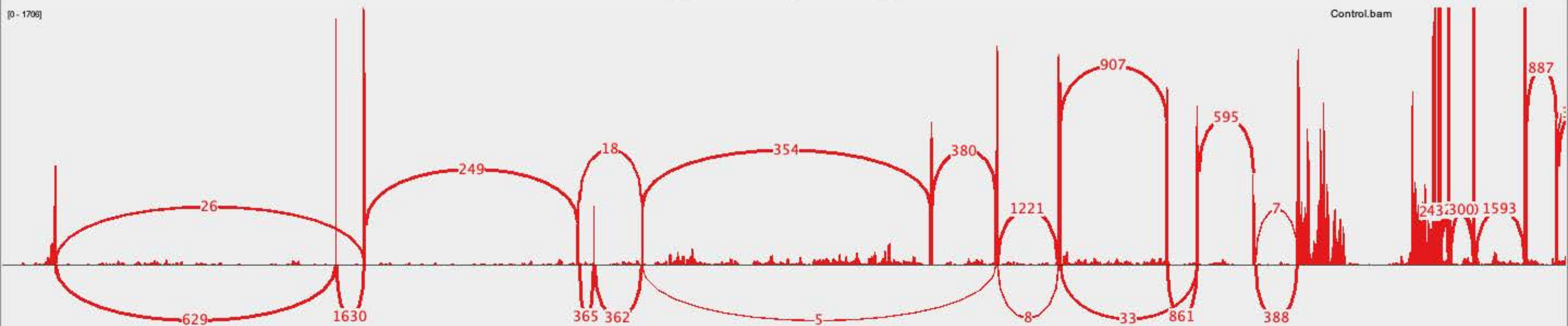

157949105

157970945

157992786

158014626

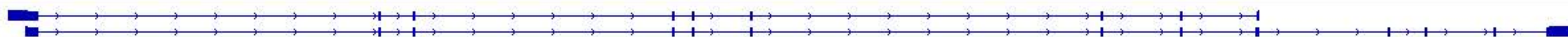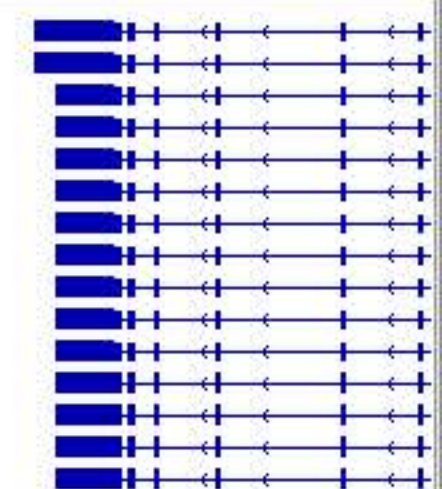



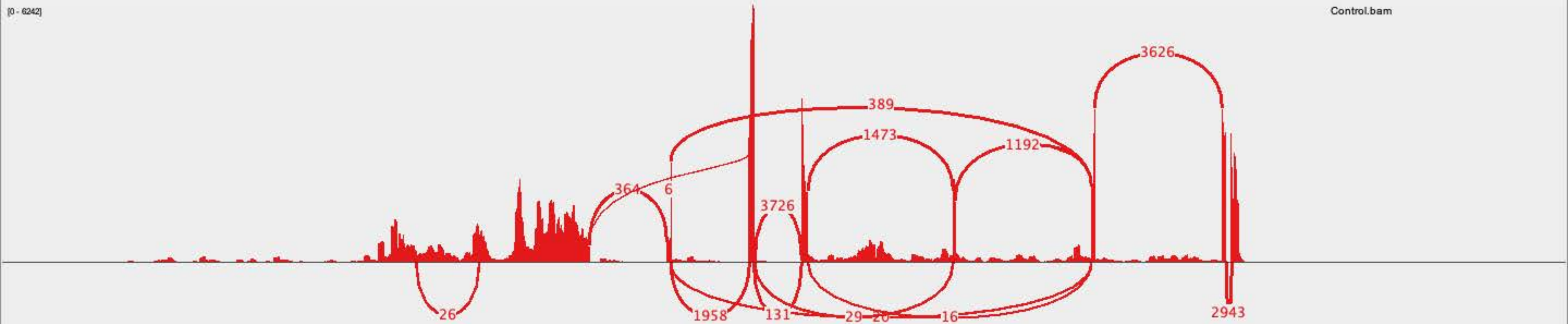

88258160 88265991 88273823 88281654

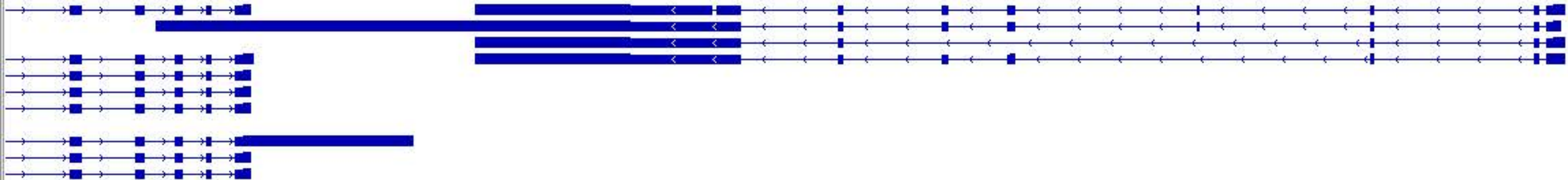

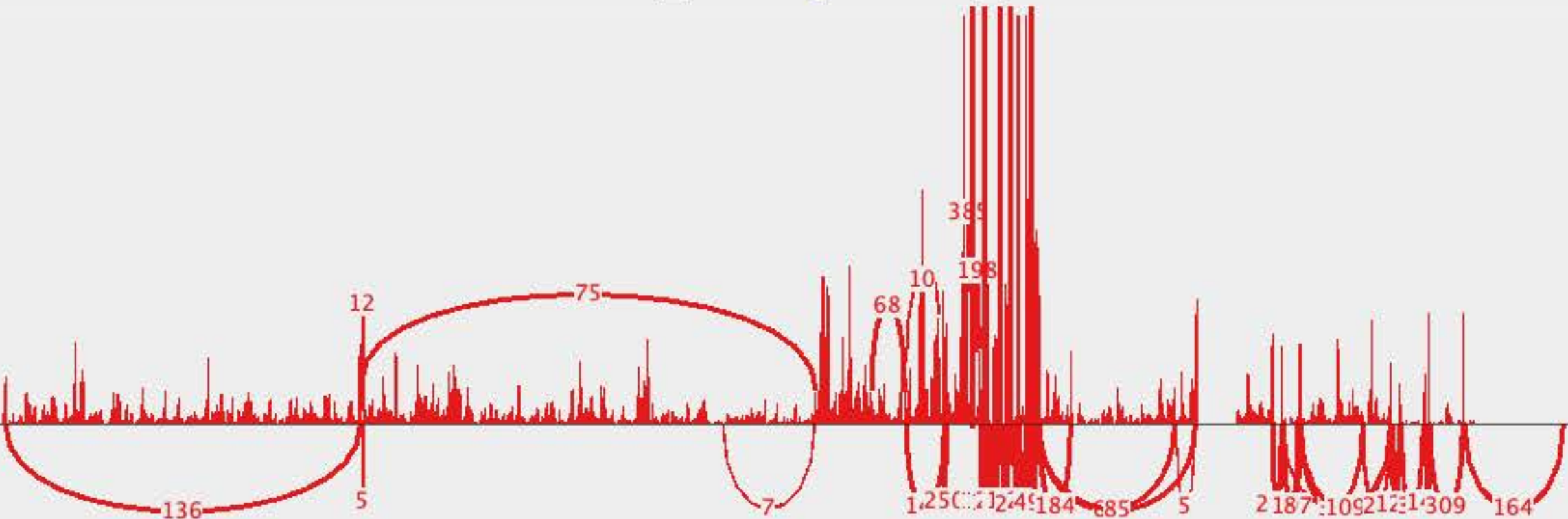

60076704 60150076 60223448 60296820

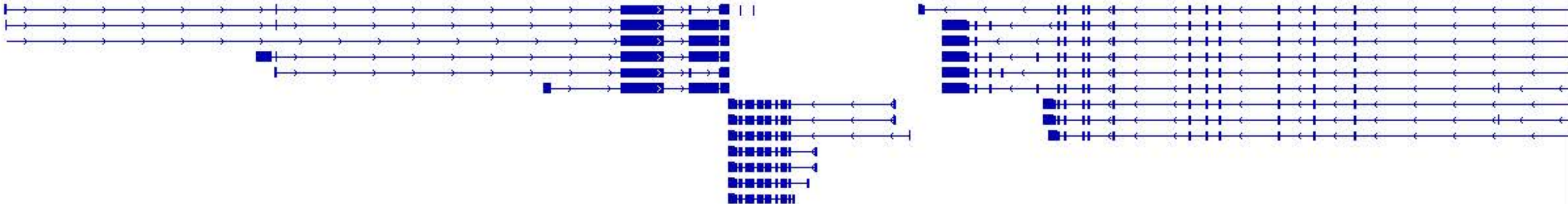

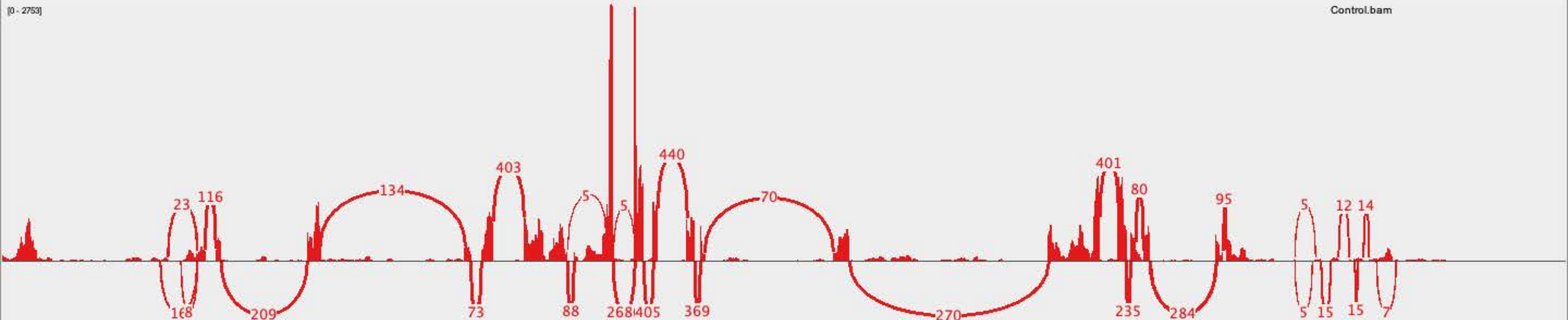

127775712 127785368 127795024 127804681

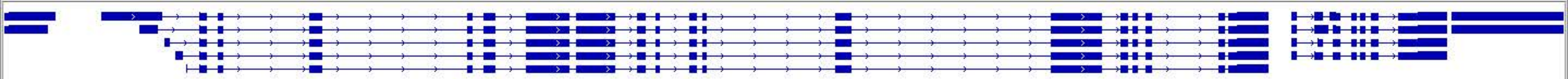

[0 - 359]

Control.bam

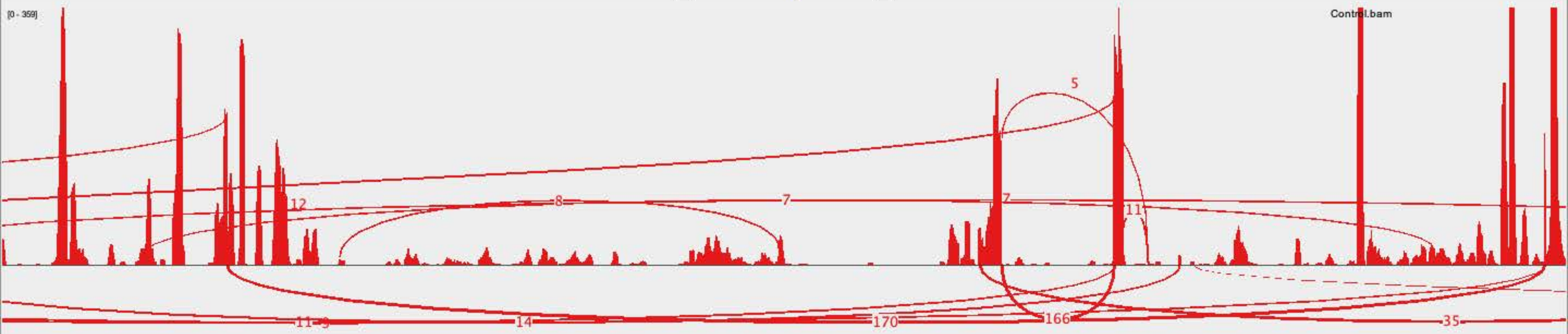

82999706

83008877

83018049

83027220

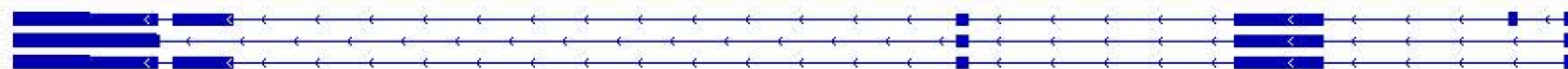



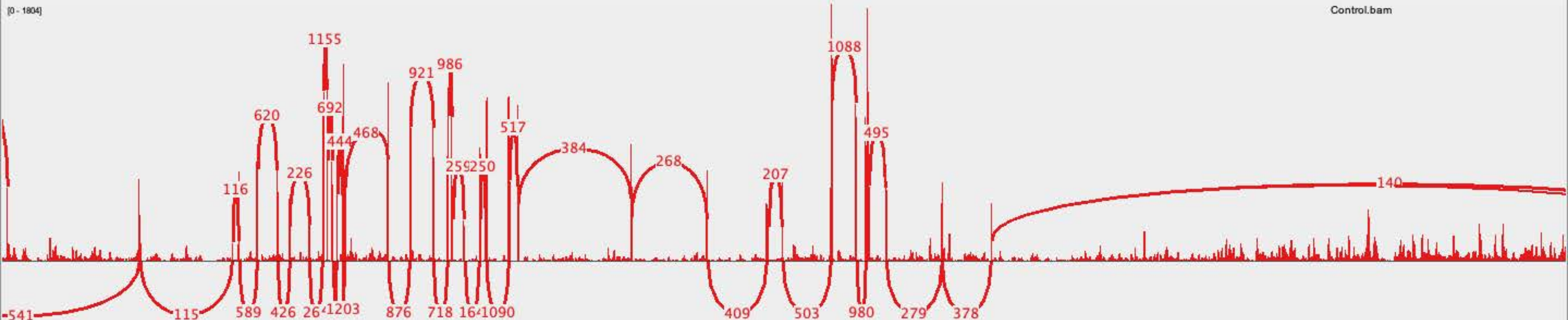

27797823 27860353 27922884 27985415

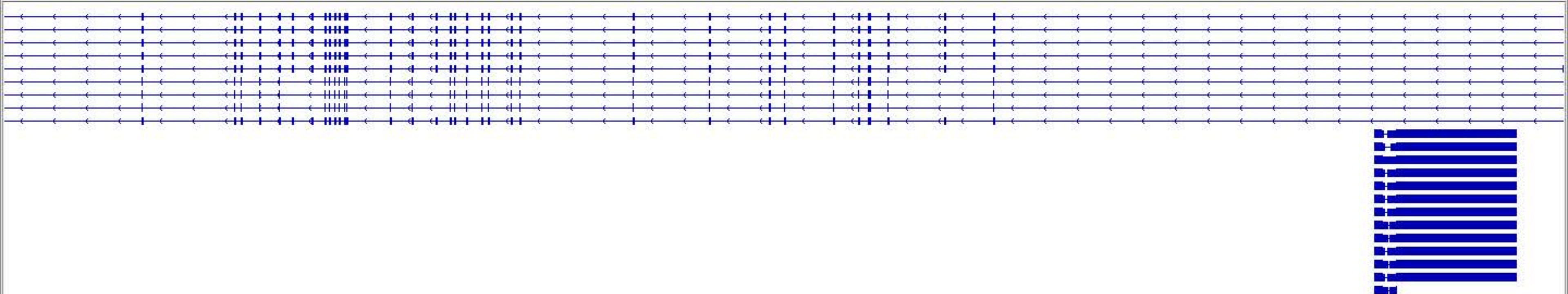

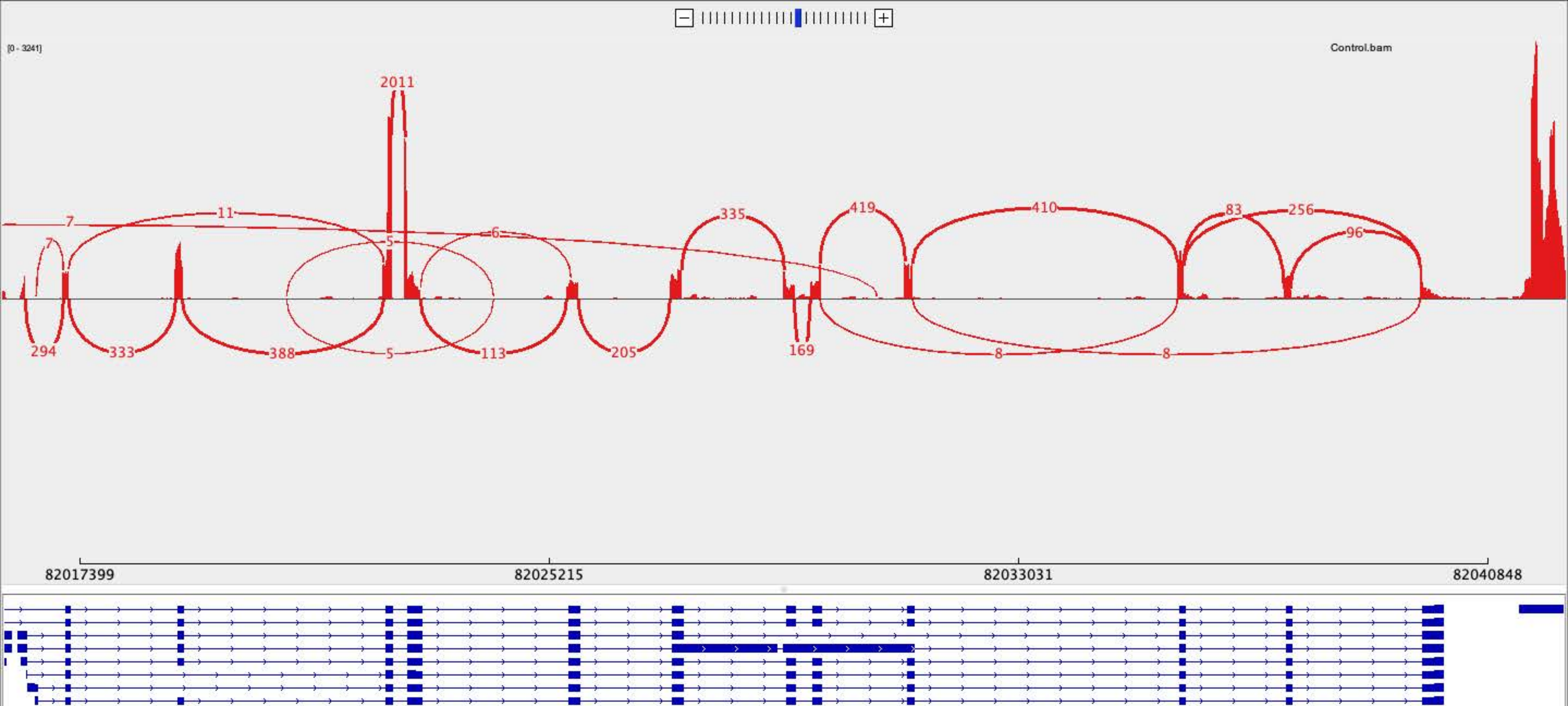

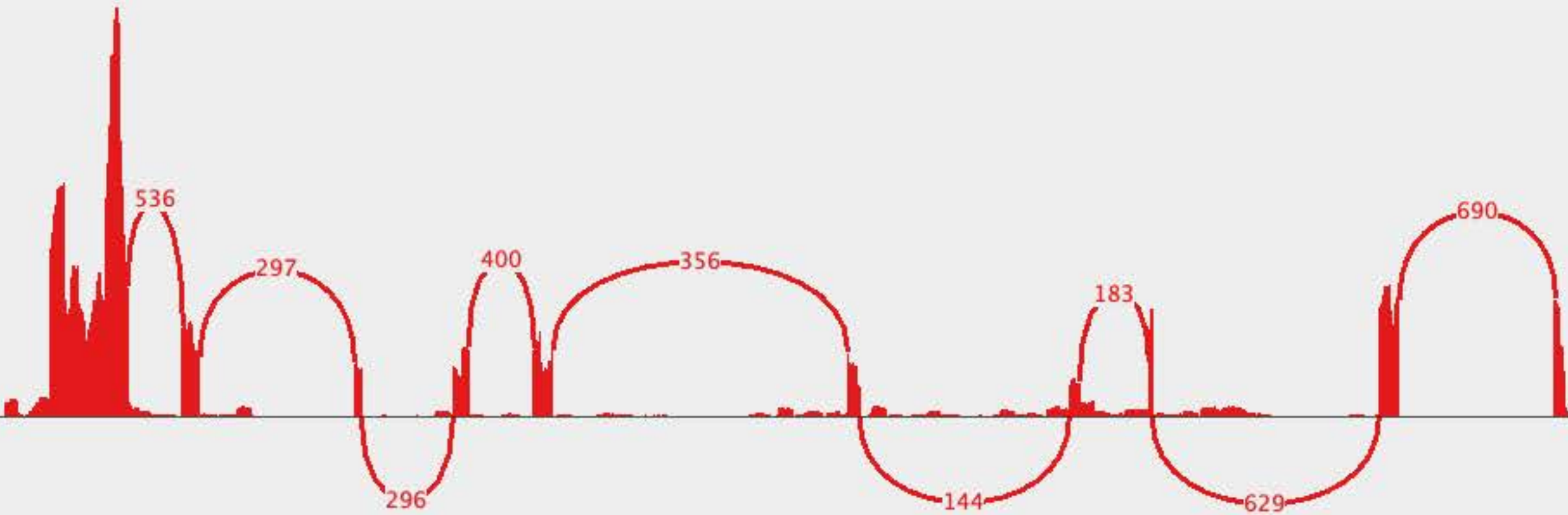

82762452 82767451 82772451 82777451

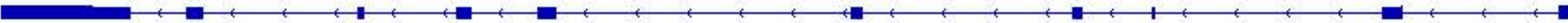

Supplement: Supplementary file 1 [file mmc1.pdf]

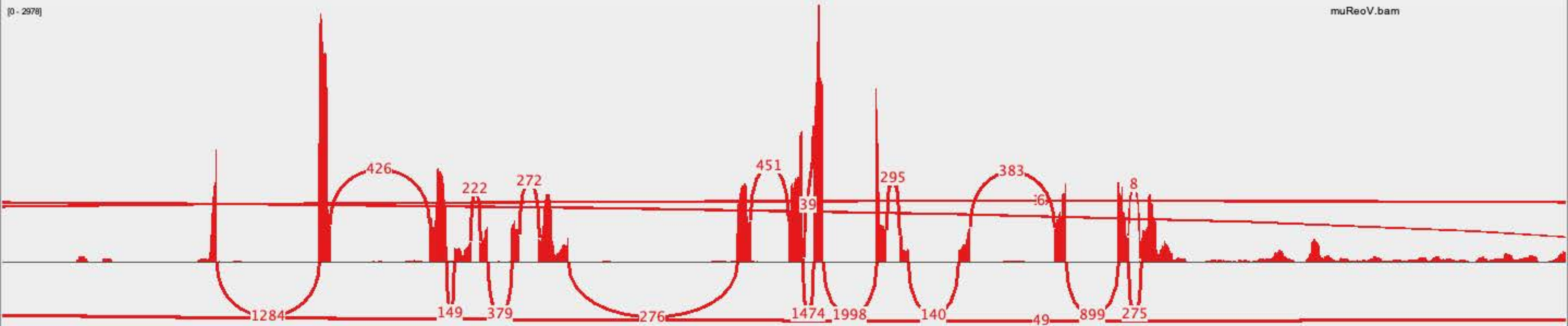

20497642

20502644

20507646

20512648

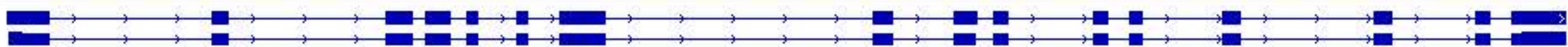

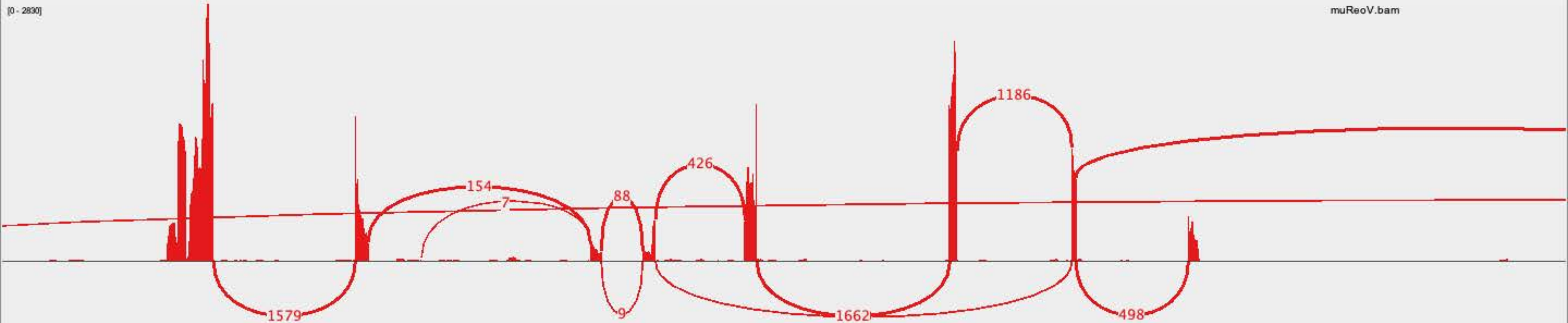

7766922

7772781

7778640

7784499

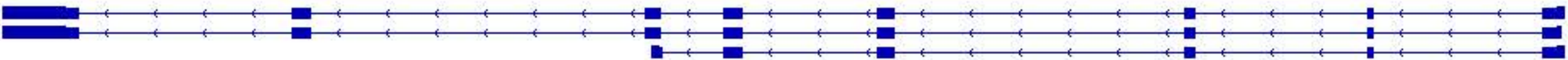

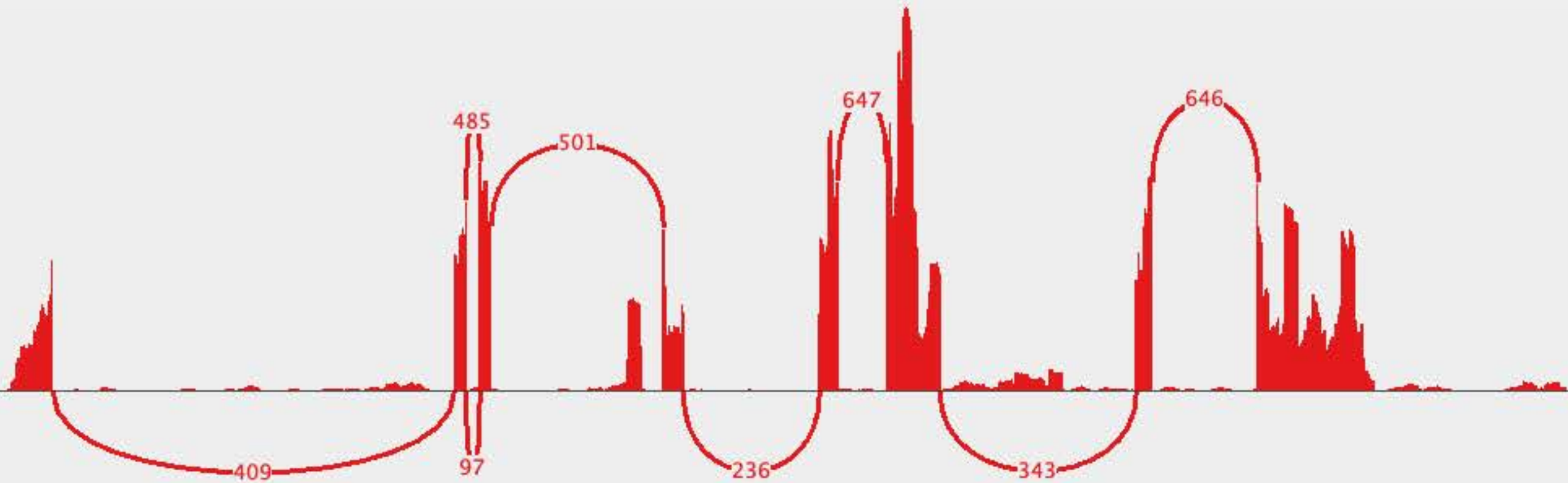

94384896 94389455 94394014 94398573

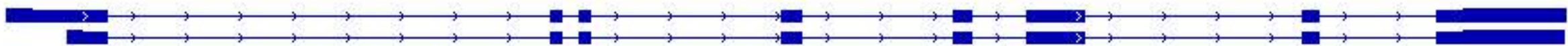

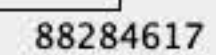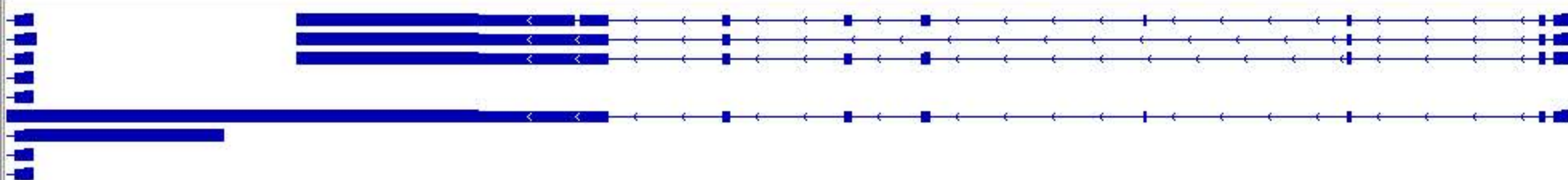

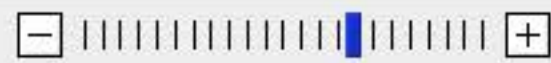

[0 - 2643]

muReoV.bam

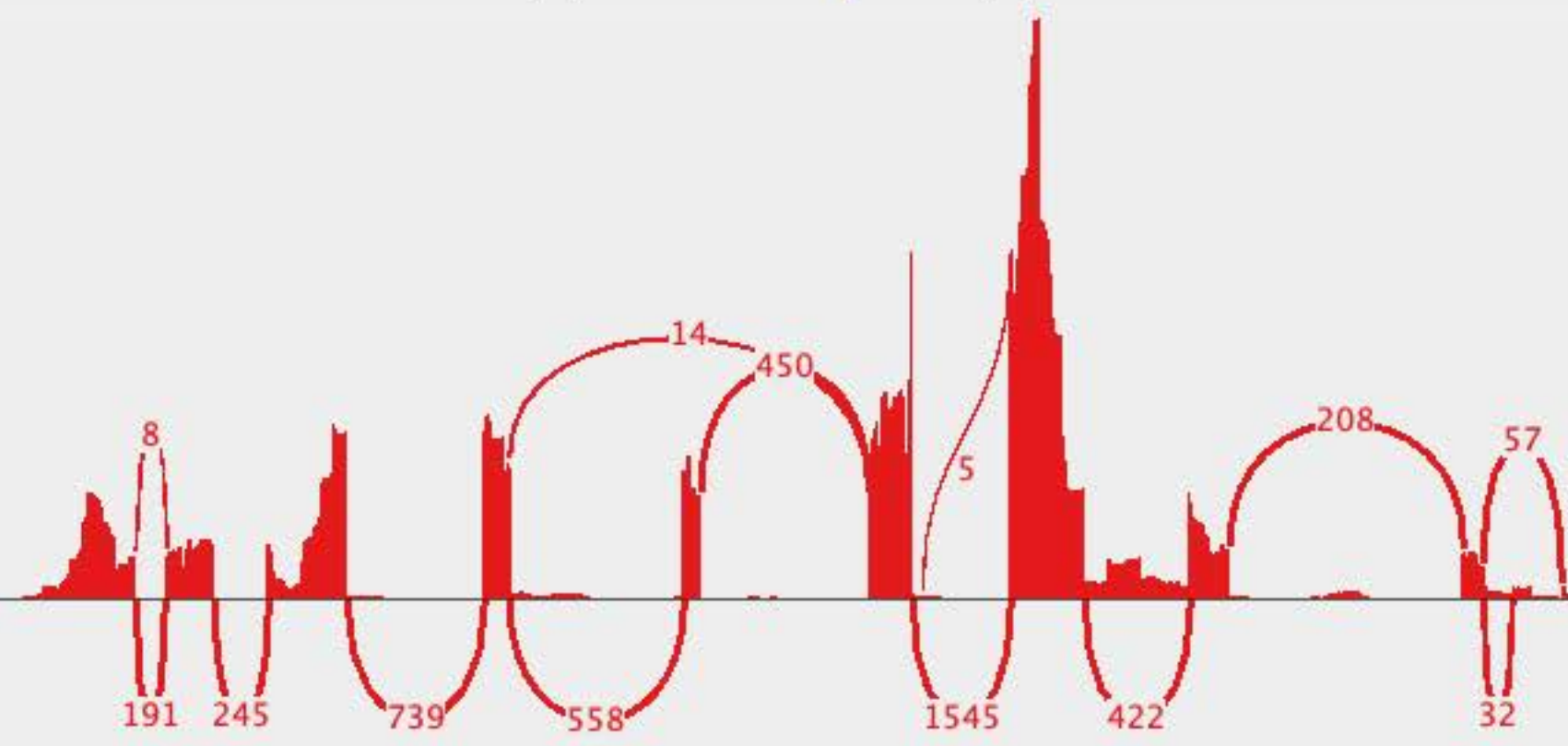

81344466 81346997 81349529 81352061

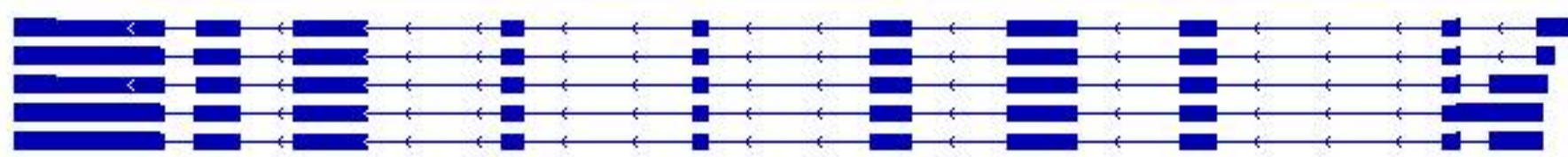

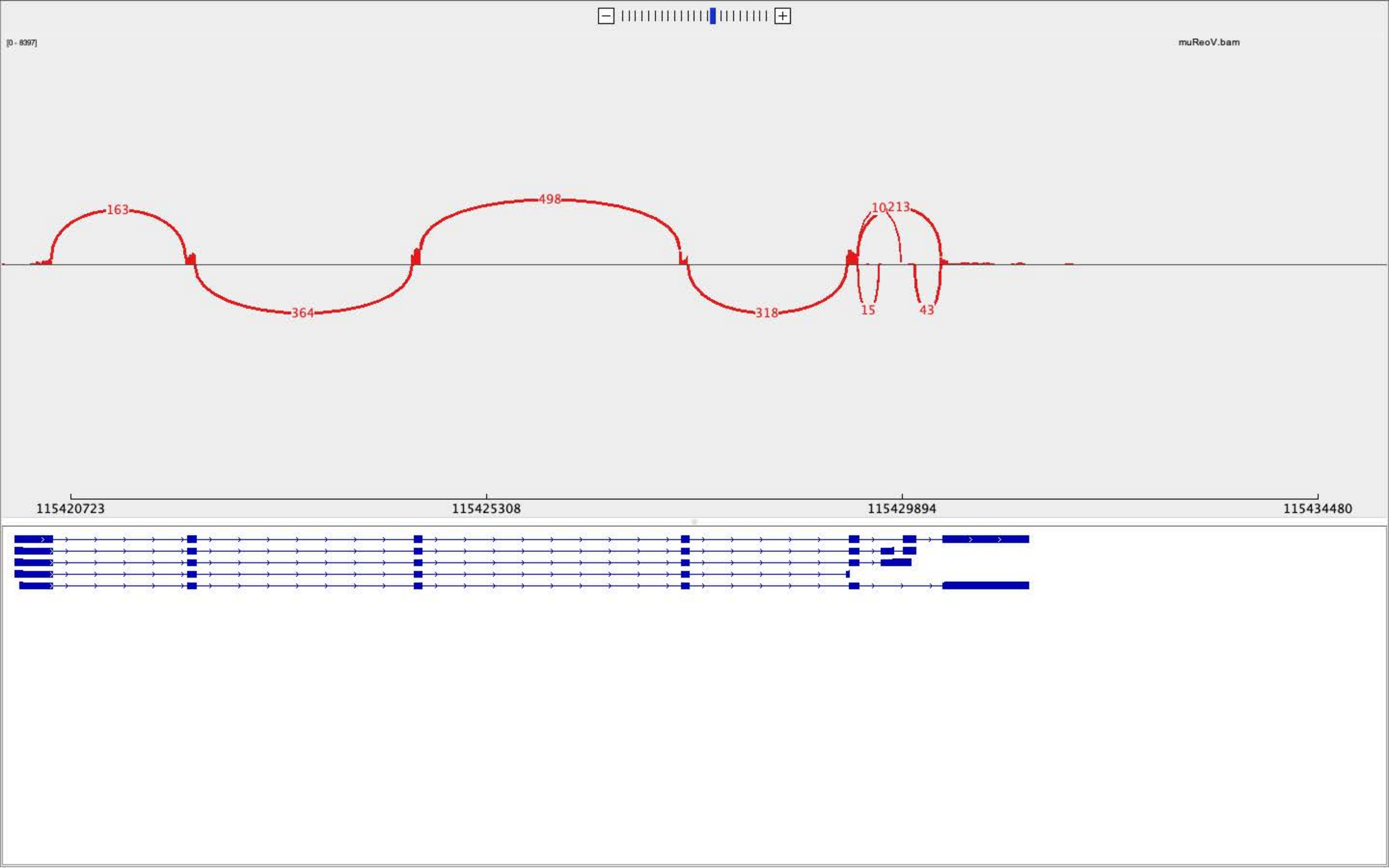

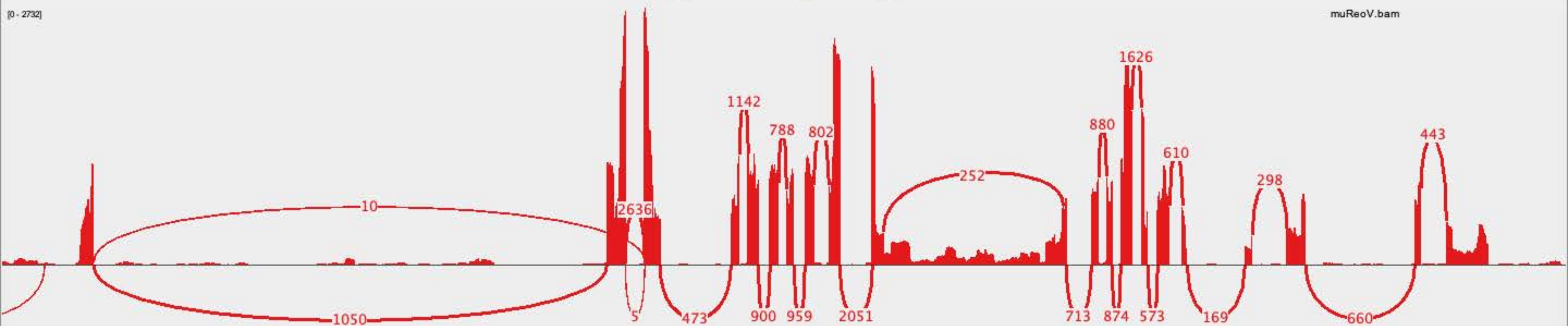

8757132

8761747

8766362

8770977

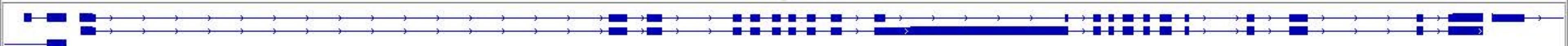

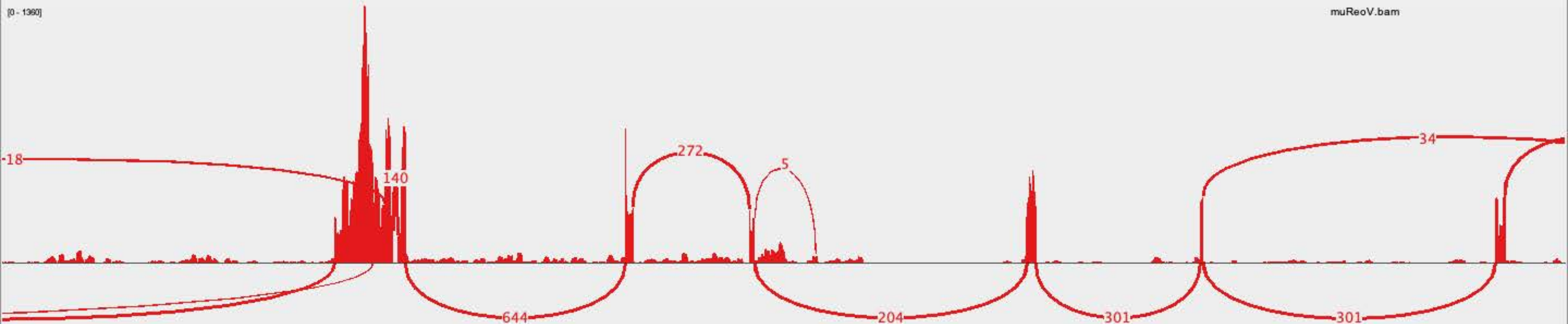

44277691

44286715

44295740

44304765

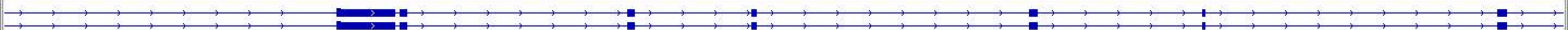

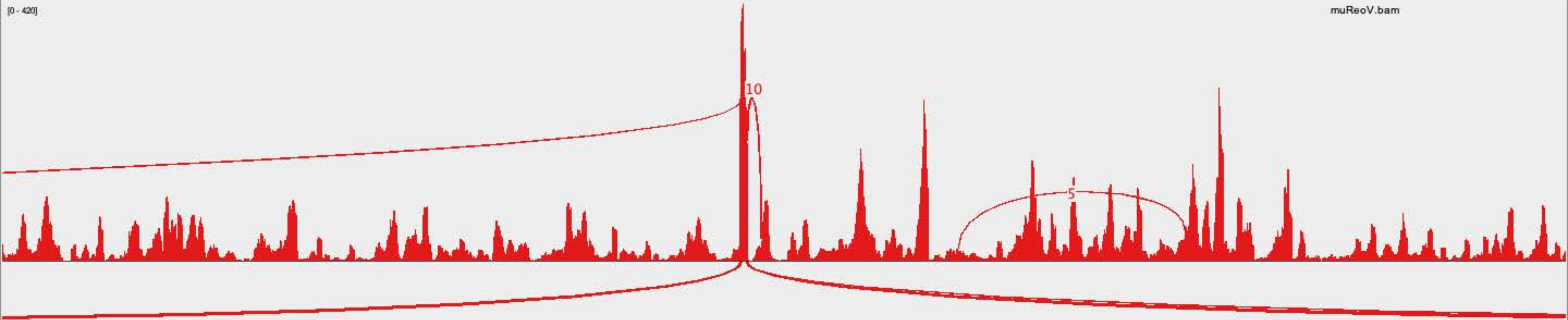

60127334 60136505 60145677 60154848

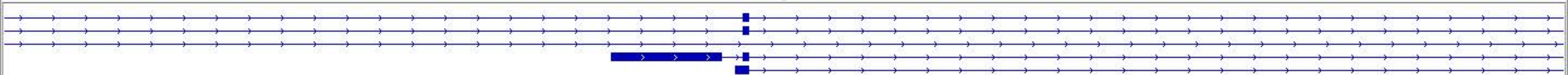

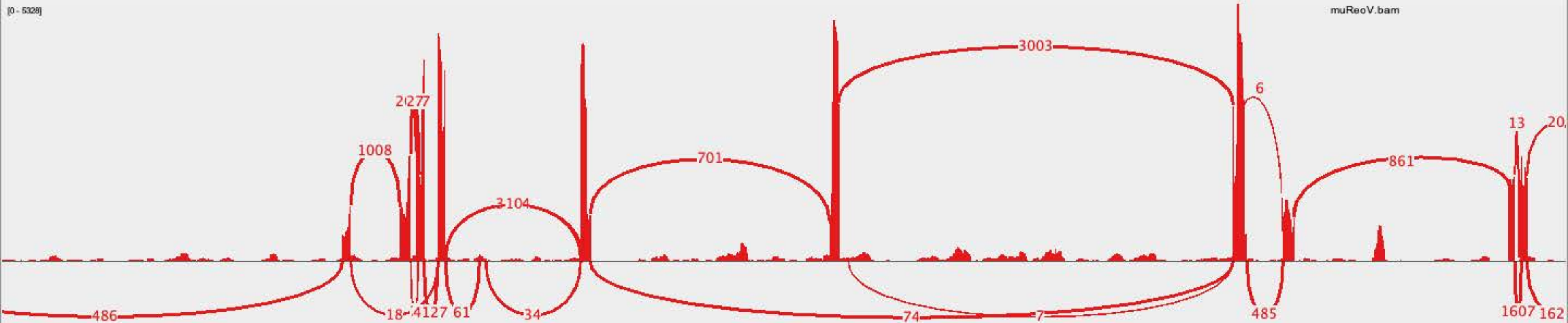

12132046 12138860 12145674 12152488

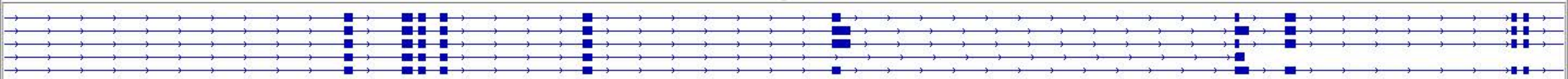

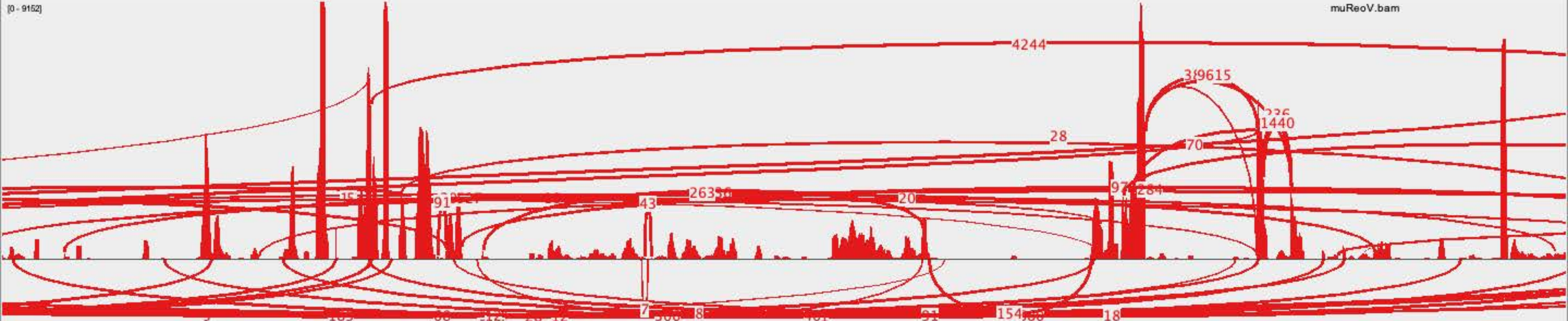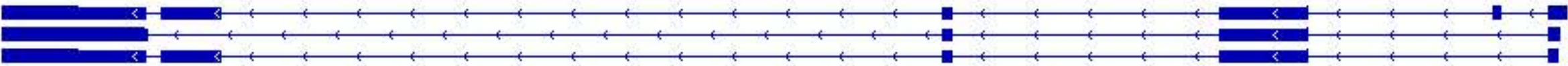

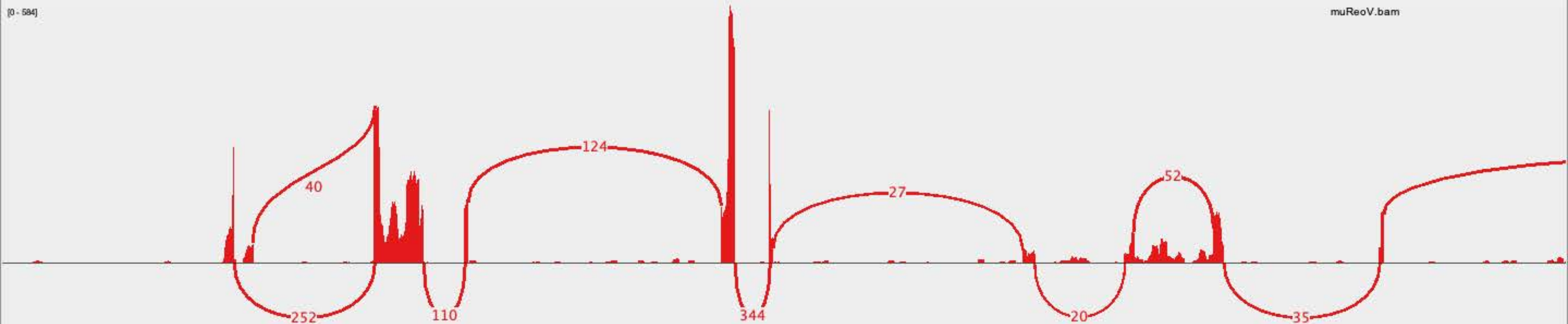

72581035

72588377

72595719

72603062

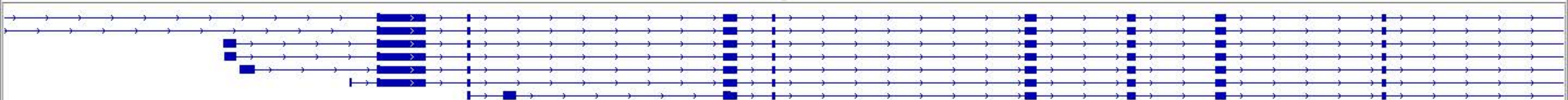

[0 - 3170]

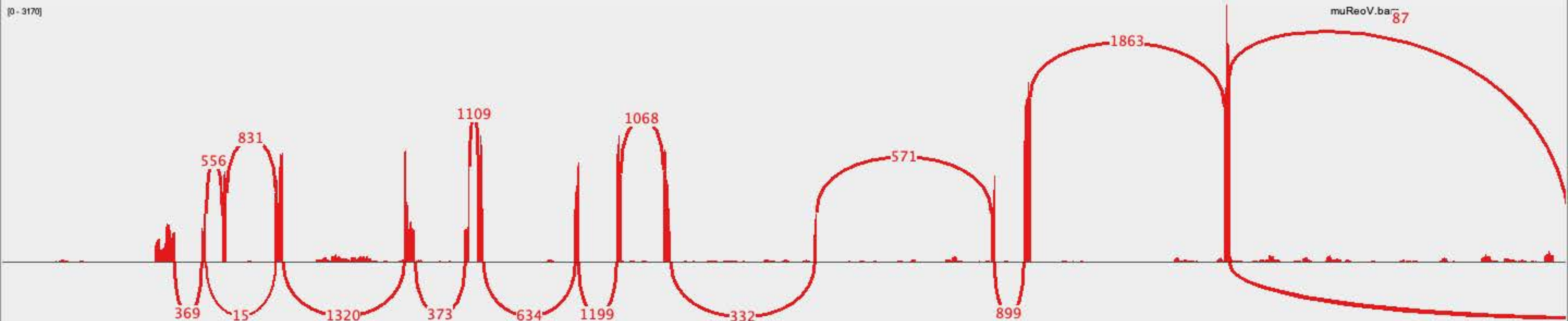

66086693

66096053

66105413

66114773

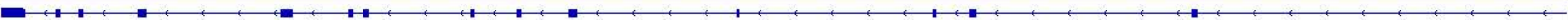

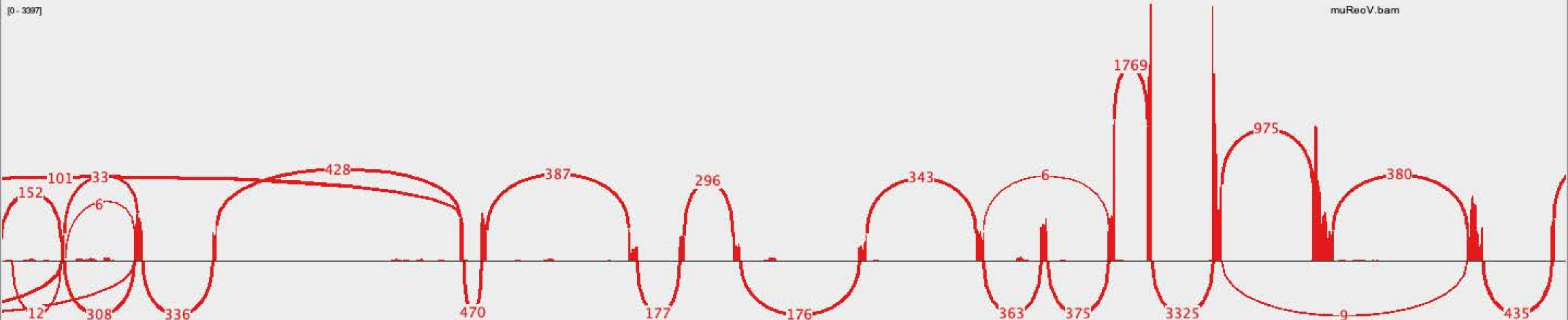

94409981 94417358 94424736 94432114

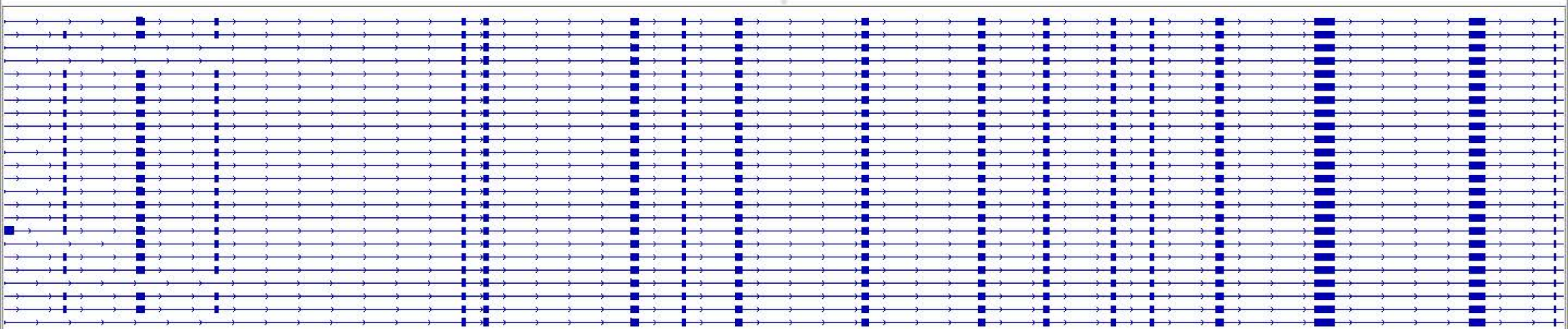

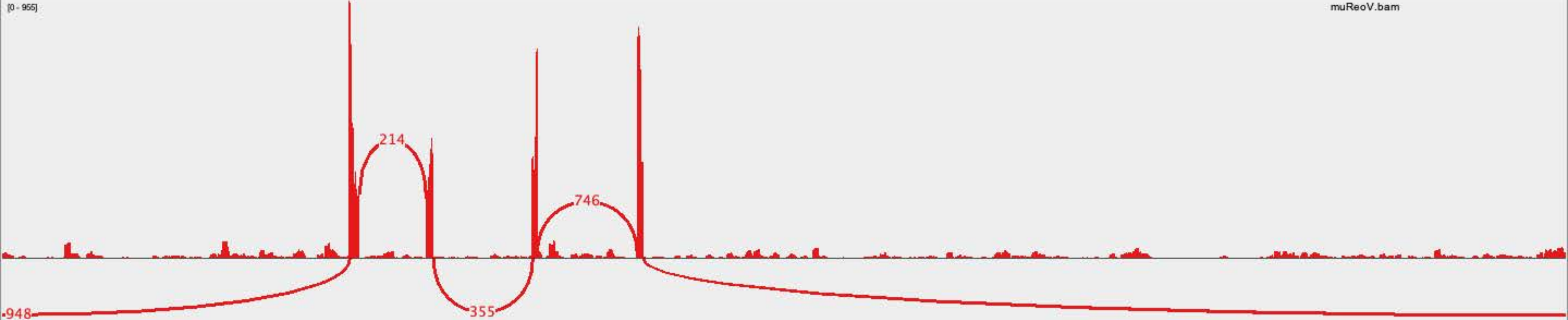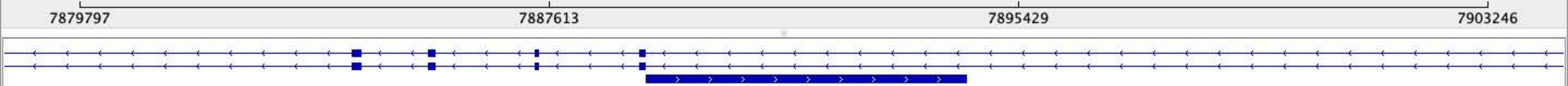

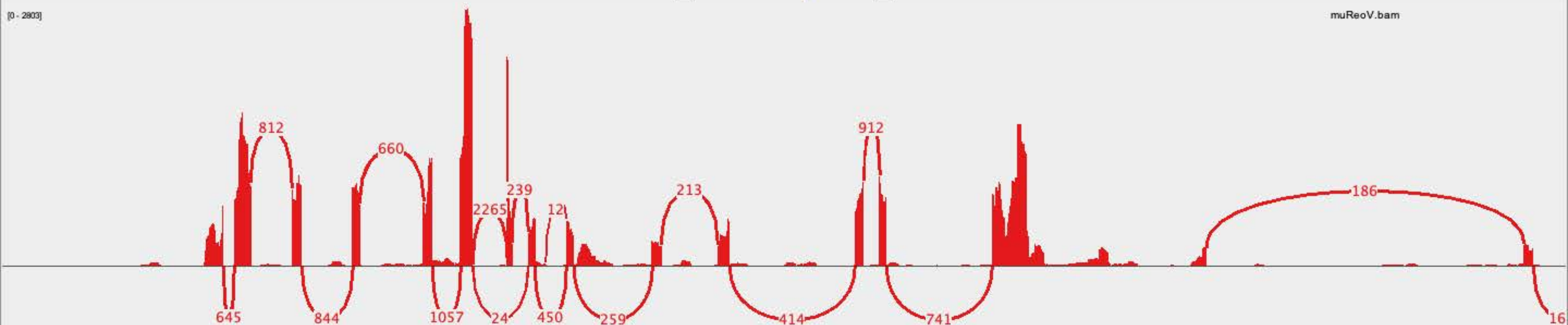

46271941

46276621

46281301

46285981

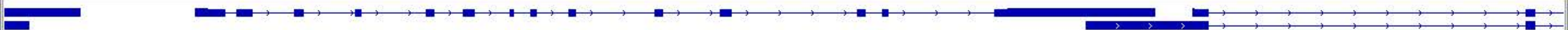

Supplement: Supplementary file 2 [file mmc2.pdf]

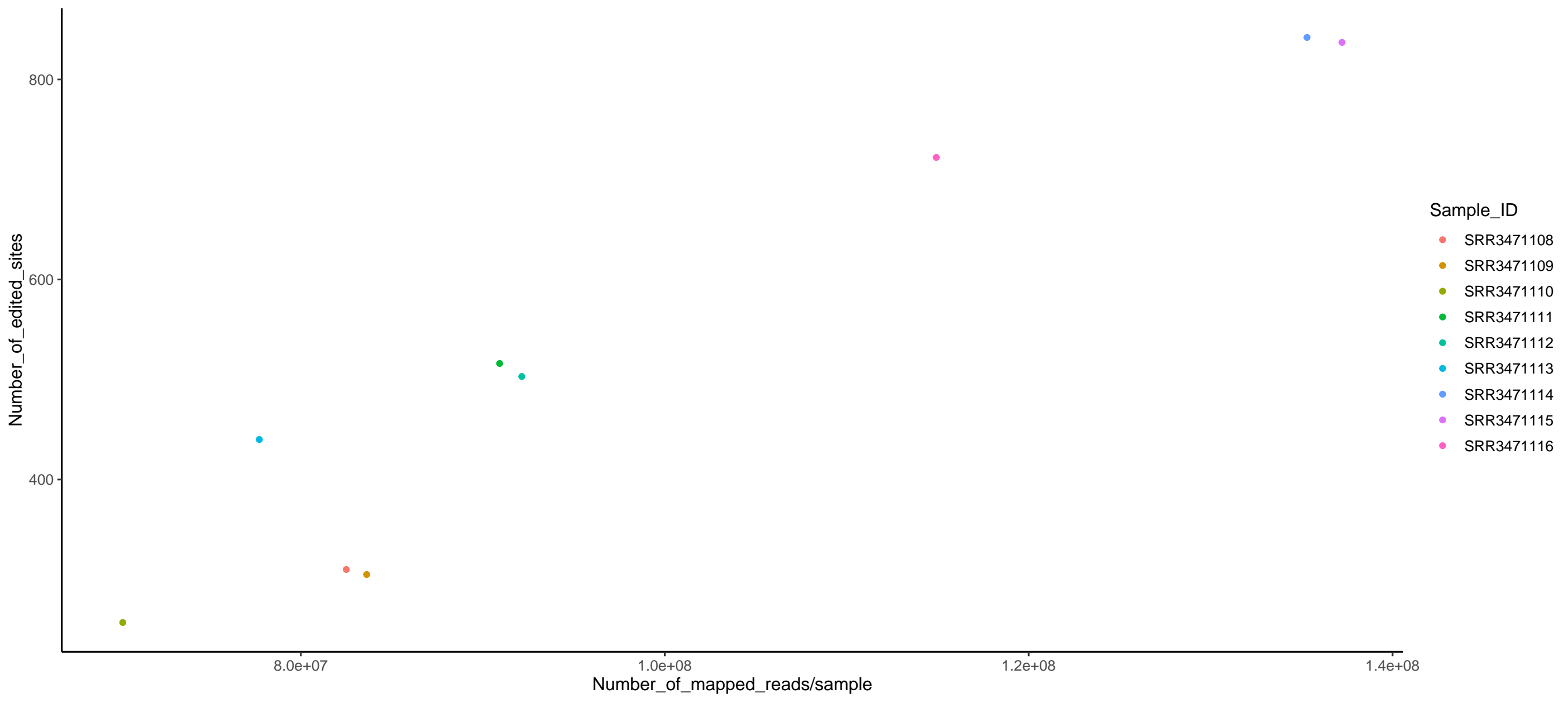

Supplement: Supplementary file 3 [file mmc3.pdf]

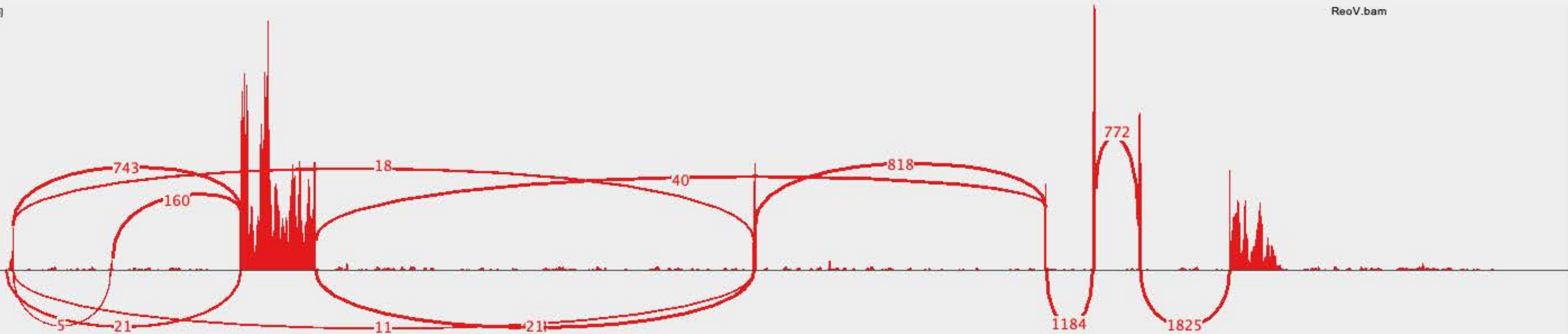

52505917 52530802 52555688 52580574

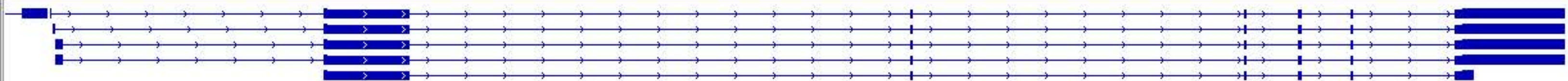

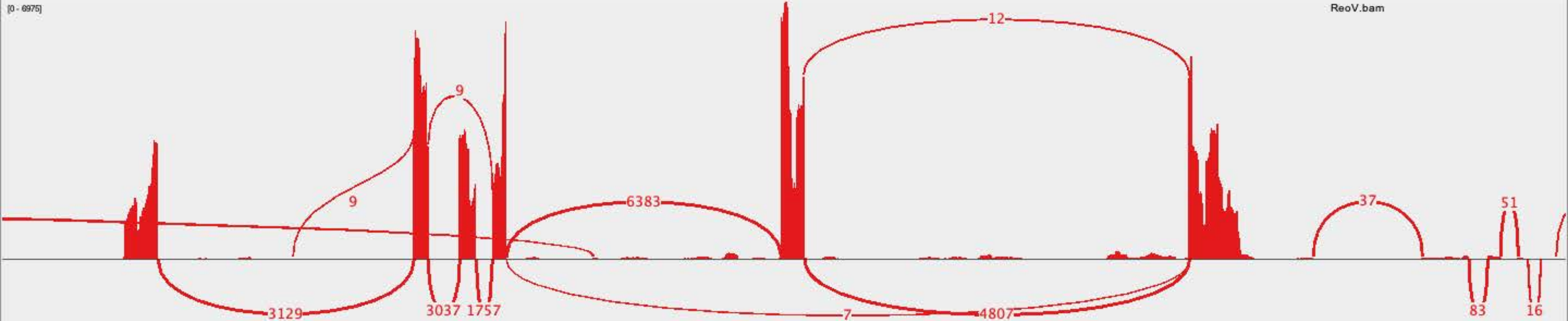

35049898

35053465

35057033

35060601

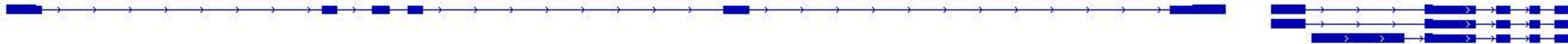

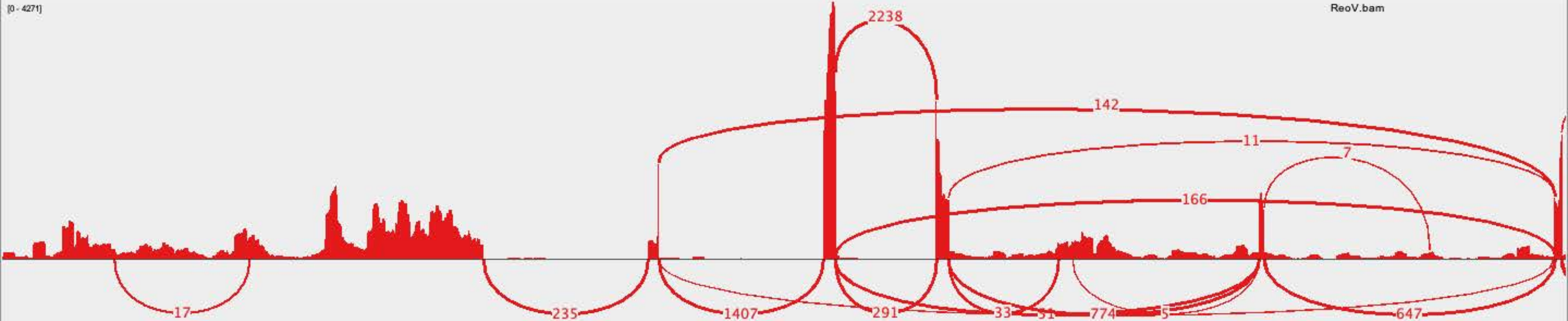

88263502 88267173 88270844 88274515

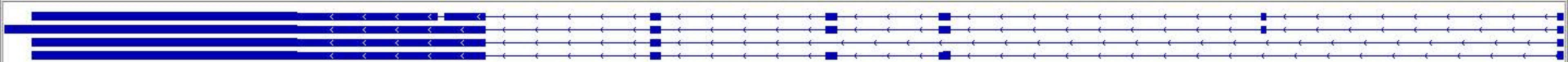

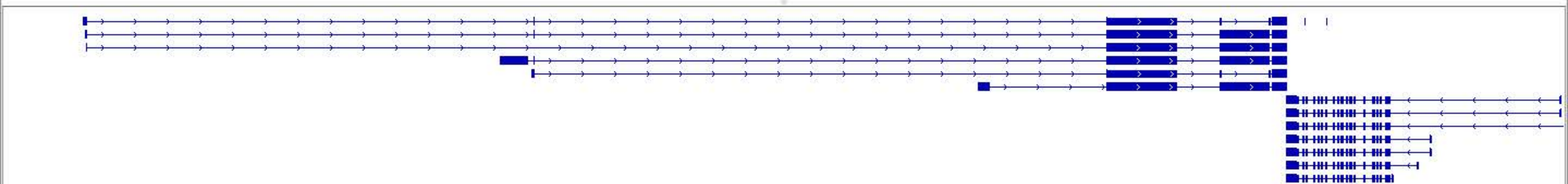

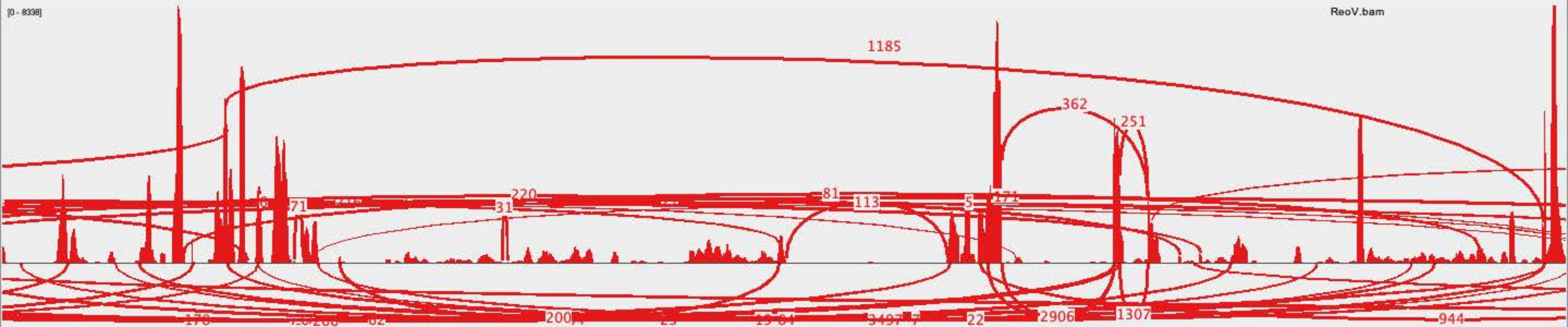

82999706

83008877

83018049

83027220

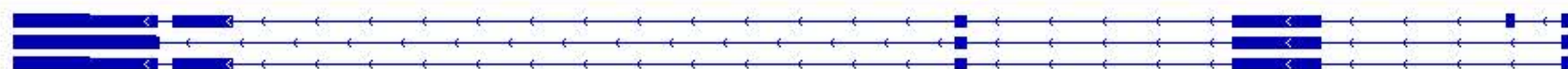

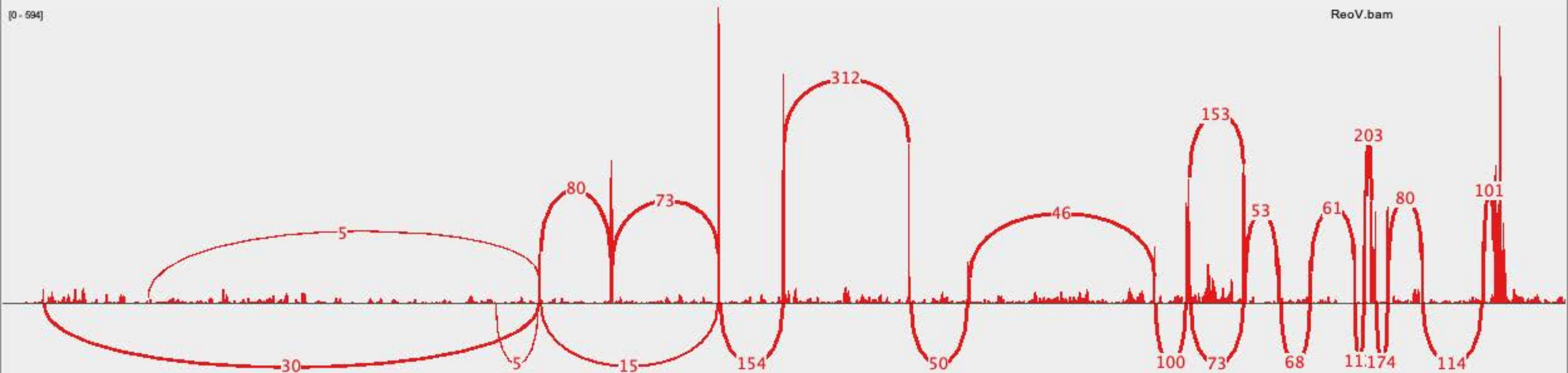

37873875

37904267

37934660

37965053

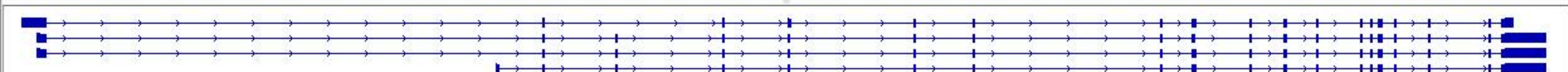

Supplement: Supplementary file 4 [file mmc4.pdf]

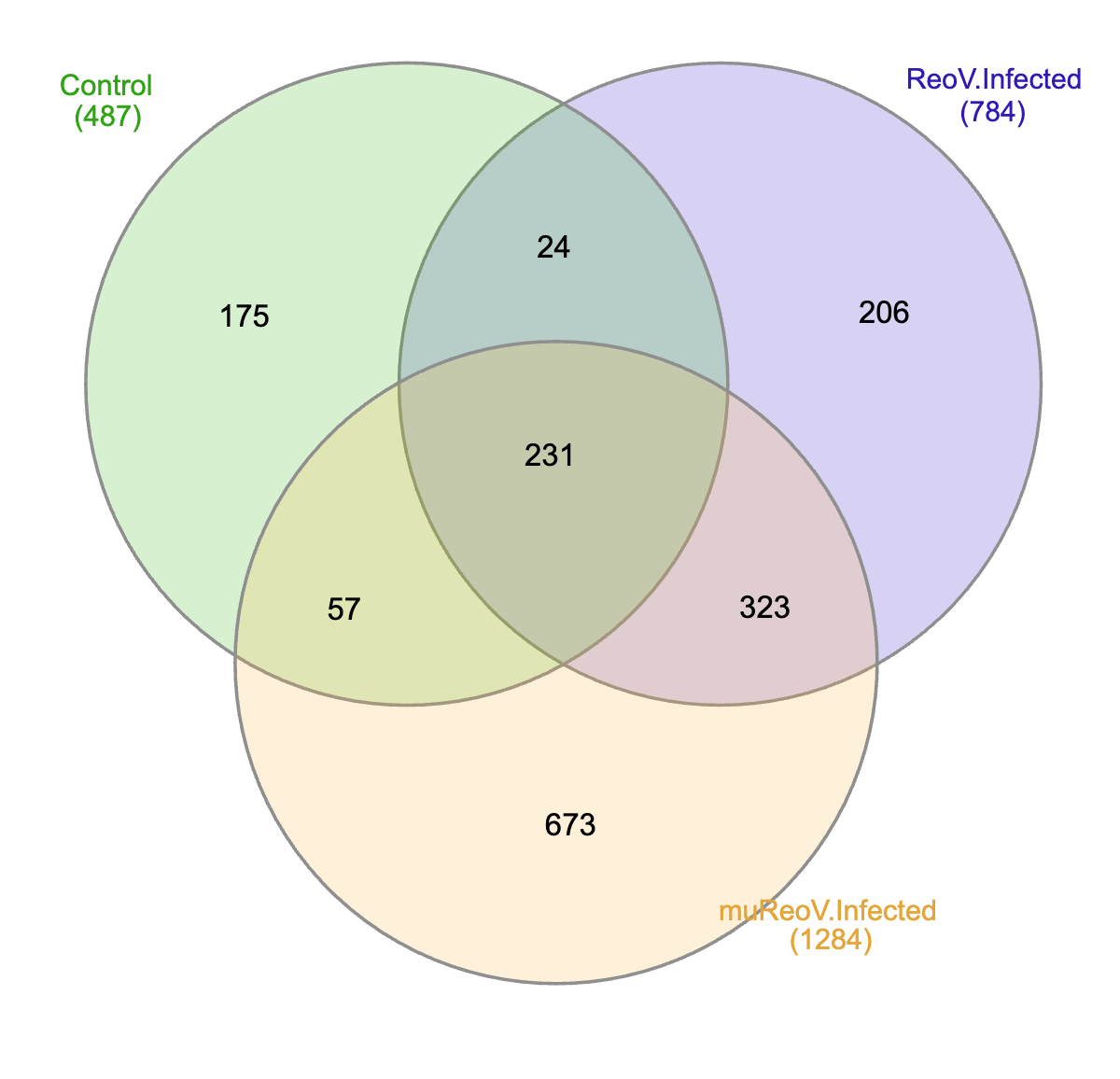

Supplement: Supplementary file 5 [file mmc5.zip › mmc5.png]

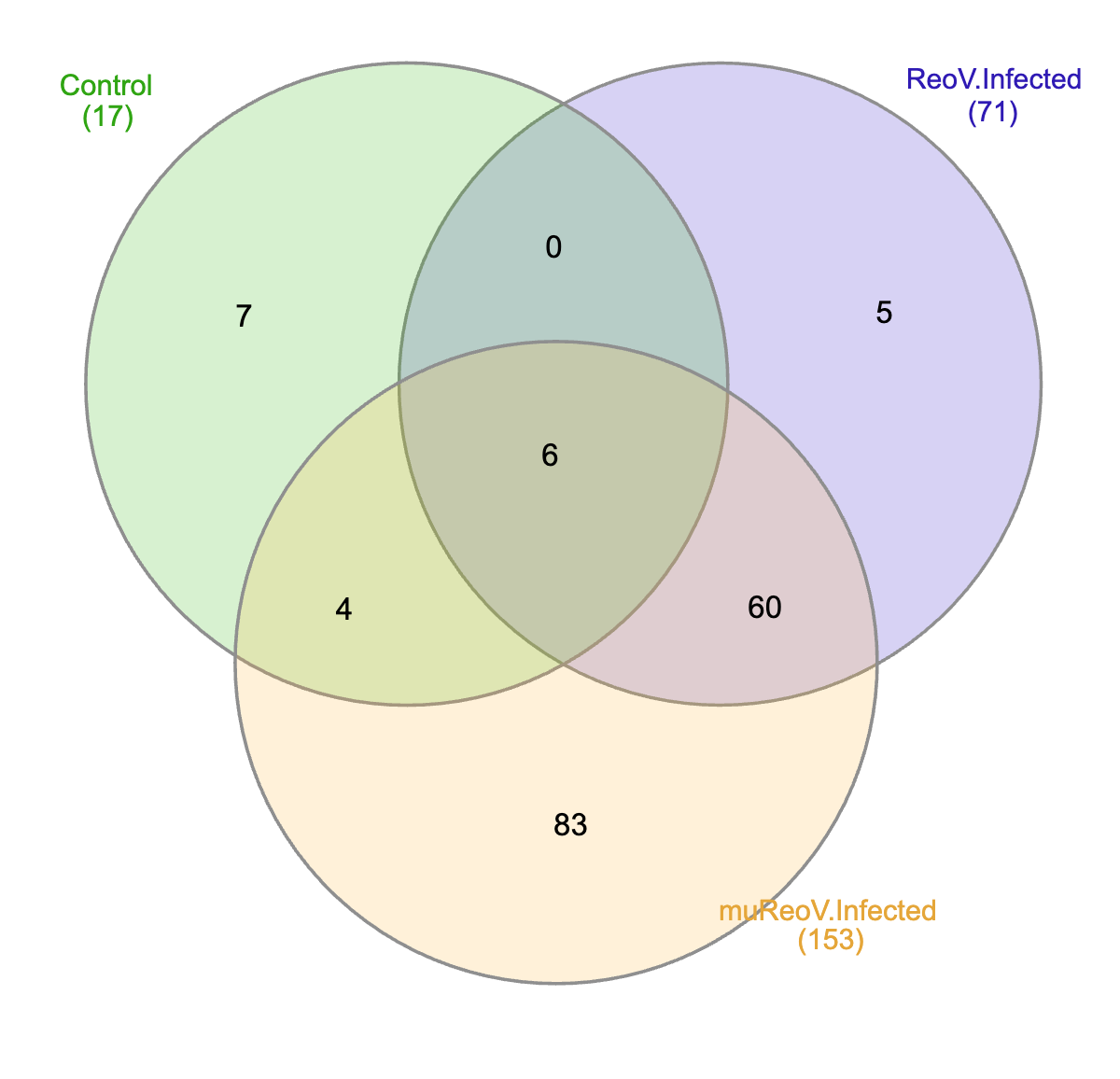

Supplement: Supplementary file 6 [file mmc6.zip › mmc6.png]
